# Students’ guide to documentation in clinical trials

**DOI:** 10.1007/s00228-022-03394-z

**Published:** 2022-09-30

**Authors:** Johannes Heck, Ann-Kathrin Rath, Katrin Wons, Carsten Schumacher, Anna Kutschenko, Nina Noltemeyer, Sarana Ulaganathan, Luca J. Voßiek, Hemme Hijma, Jeroen van Smeden, Christoph Schröder, Dirk O. Stichtenoth, Heiner Wedemeyer, Christoph Schindler, Jacobus J. Bosch

**Affiliations:** 1 Institute for Clinical Pharmacology, Hannover Medical School, Carl-Neuberg-Str. 1, 30625 Hannover, Germany; 2 Center for Clinical Trials, Hannover Medical School, Hannover, Germany; 3 Department for Gastroenterology, Hepatology and Endocrinology, Hannover Medical School, Hannover, Germany; 4 Centre for Human Drug Research, Leiden, The Netherlands; 5 Leiden University Medical Center, Leiden, The Netherlands

**Keywords:** Clinical trials, Good Clinical Practice, Medical education, Teaching, Interdisciplinarity

## Abstract

**Purpose:**

In the wake of the coronavirus disease 2019 (COVID-19) pandemic, support in clinical trials by students of human medicine and related disciplines has become of even greater importance than in pre-pandemic times. Documentation in clinical trials adheres to the principles of Good Clinical Practice (GCP), and healthcare professionals involved in the conduct of clinical trials—including students—are obliged to perform documentation in accordance with GCP principles. Unprecedented challenges have arisen with regard to the appropriate training of students as training courses in presence had largely to be suspended due to social-distancing regulations during the heyday of the COVID-19 pandemic. Therefore, novel training formats and self-study training materials for students working in clinical trials are urgently warranted.

**Methods:**

To overcome this shortcoming and to define a common quality standard, an interdisciplinary, multiprofessional (physicians, study nurses, medical students), and binational (Germany, The Netherlands) expert panel convened and devised the *Students’ guide to documentation in clinical trials*.

**Results:**

Following a brief description of the different roles in clinical trials (e.g., sponsor, (principal) investigator, monitor) and an introduction into the principles of GCP, the documentation of adverse events, concomitant medication, medical history, and quality control are comprehensively discussed. The Guide concludes with a trilingual medical dictionary (English, German, Dutch) and with recommendations of pertinent literature for further reading.

**Conclusion:**

Serving both as textbook for self-training and as (quick-) reference work for the daily routine, the Guide has specifically been designed to complement, but not to replace practical training courses for students. While primarily addressed at students of human medicine and related disciplines, the Guide can also be of high relevance and utility to other healthcare professionals involved in the conduct of clinical trials.

**Supplementary Information:**

The online version contains supplementary material available at 10.1007/s00228-022-03394-z.

In the wake of the coronavirus disease 2019 (COVID-19) pandemic, support in clinical trials by students of human medicine and related disciplines has become of even greater importance than in pre-pandemic times. Unprecedented challenges have arisen with regard to the appropriate training of students as training courses in presence had largely to be suspended due to social-distancing regulations during the heyday of the COVID-19 pandemic. Therefore, novel training formats (such as online seminars) and self-study training materials for students working in clinical trials are urgently warranted.

Documentation in clinical trials adheres to the principles of Good Clinical Practice (GCP), and healthcare professionals involved in the conduct of clinical trials—including students—are obliged to perform documentation in accordance with GCP principles [1]. Since the majority of students do not possess extensive experience in clinical trials, we have observed that the quality of their documentation fluctuates. To the best of our knowledge, comprehensive and well-referenced training material for students working in clinical trials is lacking to date.

To overcome this shortcoming and to define a common quality standard, an interdisciplinary, multiprofessional (physicians, study nurses, medical students), and binational (Germany, The Netherlands) expert panel convened and devised the *Students’ guide to documentation in clinical trials* (Supplementary Material). The guide encompasses approximately fifty pages of self-training material, divided into ten chapters. Following a brief description of the different roles in clinical trials (e.g., sponsor, (principal) investigator, monitor) and an introduction into the principles of GCP, the documentation of adverse events, concomitant medication, medical history, and quality control are comprehensively discussed. The guide concludes with a trilingual medical dictionary (English, German, Dutch) and with recommendations of pertinent literature for further reading.

Serving both as textbook for self-training and as (quick-) reference work for the daily routine, the guide has specifically been designed to complement, but not to replace practical training courses for students. While primarily addressed at students of human medicine and related disciplines, the guide can also be of high relevance and utility to other healthcare professionals involved in the conduct of clinical trials.

However, the guide is not without limitations. The guide is to a large extent based on expert opinion and has not yet been formally demonstrated to be superior to the usual standard of training. To test the guide’s usefulness in comparison with the established standard of training, our institutions are currently devising a binational randomized controlled validation study. Furthermore, the guide’s medical dictionary features only two European languages (i.e., German and Dutch) alongside English, limiting its applicability in countries in which other languages are spoken by the majority of the population. Linguistic adaptations may therefore be required in the future to allow for a more widespread application of the Guide. Notwithstanding, as nine out of ten chapters of the Guide are completely written in English, we are convinced that the guide represents a valuable resource that we would like to share with the clinical research community.

## Supplementary Information

Below is the link to the electronic supplementary material.

Supplementary file1 (DOCX 9629 KB)

## Data Availability

The data that support the findings of this study are available upon reasonable request from the corresponding author.
