# Supplementary material for: Students’ guide to documentation in clinical trials
Source: Eur J Clin Pharmacol. 2022 Sep 30;78(11):1861–2. doi: 10.1007/s00228-022-03394-z (PMC9524727; doi:10.1007/s00228-022-03394-z)
Supplement: Supplementary file 1 — Supplementary file1 (DOCX 9629 KB) [file 228_2022_3394_MOESM1_ESM.docx]

| 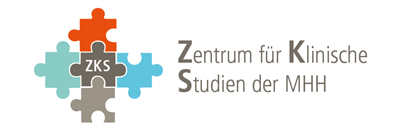 | 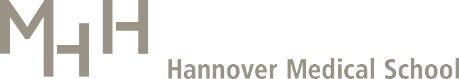 | 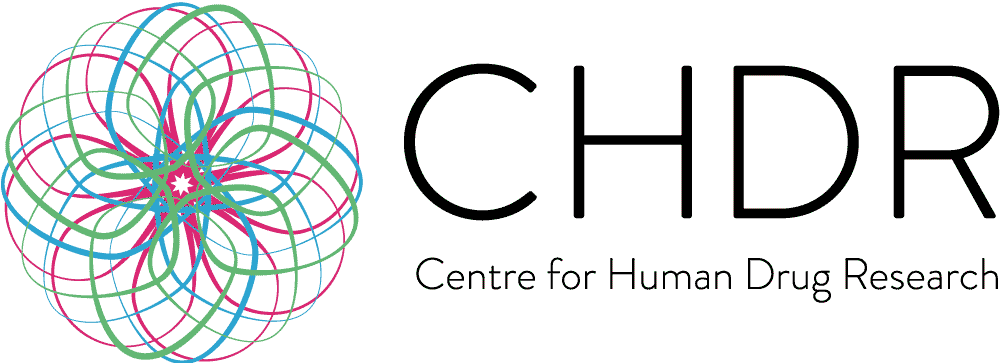 |
| --- | --- | --- |

**Students’ Guide to Documentation in Clinical Trials**

Johannes Heck^1,2*^, Ann-Kathrin Rath^2,3*^, Katrin Wons^2^, Carsten Schumacher^2^, Anna Kutschenko^2^, Nina Noltemeyer^2^, Sarana Ulaganathan^2^, Luca J. Voßiek^2^, Hemme Hijma^4,5^, Jeroen van Smeden^4,5^, Christoph Schröder^1^, Dirk O. Stichtenoth^1^, Heiner Wedemeyer^3^, Christoph Schindler^2^, Jacobus J. Bosch^2,4,5^

^1^Institute for Clinical Pharmacology, Hannover Medical School, Hannover, Germany

^2^Center for Clinical Trials, Hannover Medical School, Hannover, Germany

^3^Department for Gastroenterology, Hepatology and Endocrinology, Hannover Medical School, Hannover, Germany

^4^Centre for Human Drug Research, Leiden, The Netherlands

^5^Leiden University Medical Center, Leiden, The Netherlands

^*^Johannes Heck and Ann-Kathrin Rath are joint first authors.

**Contents**

Figures V

Tables VI

Abbreviations VII

1 Document history 1

2 Introduction 2

3 Roles in clinical trials 3

4 General considerations and principles of Good Clinical Practice 5

4.1 Writing utensils and text color 5

4.2 Date formatting 5

4.3 Handling of empty fields 6

4.4 Changes and corrections 6

4.5 Worksheet check 6

5 Adverse events 8

5.1 Definition of an adverse event 8

5.2 Definition of a serious adverse event 9

5.3 Seriousness versus severity 10

5.4 Common Terminology Criteria for Adverse Events 10

5.5 Definition of an adverse event of special interest 11

5.5.1 Coronavirus disease 2019-related adverse events of special interest 12

5.5.2 Immune-related adverse events 12

5.6 Causality assessment 13

5.7 Definition of a suspected unexpected serious adverse reaction 13

5.8 Documentation of adverse events 13

5.8.1 Principle of parsimony 13

5.8.2 Examples of adverse event documentation 14

6 Concomitant medication 17

6.1 Concomitant medication terminology 17

6.2 Posology 18

6.2.1 Leading and trailing zeros 18

6.2.2 Dosages in micrograms 19

6.3 Routes of administration 19

6.3.1 Enteral administration 20

6.3.1.1 Oral (*per os*, p.o., PO) 20

6.3.1.2 Sublingual (s.l., SL) 20

6.3.1.3 Buccal 21

6.3.1.4 Rectal (*per rectum*, p.r., PR) 21

6.3.1.5 Other enteral routes of administration 21

6.3.2 Parenteral administration 21

6.3.2.1 Intravenous (i.v., IV) 21

6.3.2.2 Intramuscular (i.m., IM) 22

6.3.2.3 Subcutaneous (s.c., SC) 22

6.3.2.4 Intradermal (i.d., ID) 22

6.3.2.5 Inhalative (*per inhalationem*, p.i., PI) 22

6.3.2.6 Intranasal (i.n., IN) 23

6.3.2.7 Topical (top., TOP) 23

6.3.2.8 Transdermal (t.d., TD) 23

6.3.2.9 Intralesional 24

6.3.2.10 Ophthalmic/ocular 24

6.3.2.11 Intravitreal 24

6.3.2.12 Auricular/otic 24

6.3.2.13 Vaginal 25

6.3.2.14 Intrathecal 25

6.3.2.15 Other parenteral routes of administration 25

6.4 Drug application frequencies 25

6.5 Non-prescription medicines 26

6.6 Examples of concomitant medication documentation 27

7 Medical history 30

7.1 Documentation of medical history 31

8 Quality control 33

8.1 Quality control in clinical trials 33

8.1.1 Quality control via monitors 33

8.1.2 Internal quality control 33

8.1.2.1 Worksheets 33

8.1.2.2 Cross-checking 34

8.1.2.3 Quality management 34

8.1.3 External quality control 34

8.1.3.1 Automated data queries 34

8.1.3.2 Data management 35

8.1.3.3 Medical monitor 35

8.1.3.4 Data and safety monitoring board 35

8.1.3.5 Update on suspected unexpected serious adverse reactions 35

8.1.3.6 Audits 35

8.1.3.7 Inspections 36

8.2 Quality control of the Students’ Guide 36

8.2.1 Version control 36

8.2.2 Effective date 37

8.2.3 Document history 37

8.2.4 Page numbering 37

8.2.5 Interdisciplinarity 37

8.2.6 Multiprofessionality 38

8.2.7 Internationality 38

8.2.8 Digitalization 39

8.2.9 Educational activities 39

9 Medical dictionary German–English–Dutch 40

10 References 45

# Figures

Fig. 1: Use of overarching diagnoses in lieu of single symptoms 14

Fig. 2: Updating the adverse events log 15

Fig. 3: Documentation of a serious adverse event and documentation of an accident with ensuing injury 16

Fig. 4: Documentation of concomitant medication—first example 27

Fig. 5: Documentation of concomitant medication—second example 28

Fig. 6: Documentation of concomitant medication—third example 29

Fig. 7: Example of a complex medical history 31

# Tables

Table 1: Description of roles in clinical trials 3

Table 2: Date formatting 5

Table 3: Common Terminology Criteria for Adverse Events severity grading 11

Table 4: Concomitant medication terminology 18

Table 5: Leading and trailing zeros in medication dosages 19

Table 6: Abbreviations of drug application frequencies, their Latin origin, their meaning, and corresponding colloquial English equivalents 26

# Abbreviations

| ABC | Dummy variable |
| --- | --- |
| AE | Adverse event |
| AESI | Adverse event of special interest |
| ACE | Angiotensin-converting enzyme |
| approx. | Approximately |
| AR | Adverse reaction |
| BfArM | *Bundesinstitut für Arzneimittel und Medizinprodukte* (Federal Institute for Drugs and Medical Devices) |
| ca. | Circa |
| CEPI | Coalition for Epidemic Preparedness Innovation |
| CHDR | Centre for Human Drug Research |
| cm | Centimeter |
| ConMed | Concomitant medication |
| COPD | Chronic obstructive pulmonary disease |
| COVID-19 | Coronavirus disease 2019 |
| CRF | Case report form |
| CRO | Contract research organization |
| CT-3 | Detailed guidance on the collection, verification and presentation of adverse event/reaction reports arising from clinical trials on medicinal products for human use |
| CTCAE | Common Terminology Criteria for Adverse Events |
| CTLA-4 | Cytotoxic T-lymphocyte antigen 4 |
| d | Day |
| DM | Data management |
| DMC | Data monitoring committee |
| DSMB | Data and safety monitoring board |
| eCRF | Electronic case report form |
| e.g. | For example (abbreviation from Latin *exempli gratia*) |
| EMA | European Medicines Agency |
| EU | European Union |
| FDA | Food and Drug Administration |
| Fig. | Figure |
| FWJ | *Freiwilliges Wissenschaftliches Jahr* (voluntary scientific year) |
| g | Gram |
| GCP | Good Clinical Practice |
| GERD | Gastroesophageal reflux disease |
| IB | Investigator’s brochure |
| ICD | Implantable cardioverter defibrillator |
| ICF | Informed consent form |
| ICH | International Council for Harmonisation of Technical Requirements for Registration of Pharmaceuticals for Human Use |
| ID | Identification number |
| i.d., ID | Intradermal |
| i.e. | That is to say (abbreviation from Latin *id est*) |
| IGJ | *Inspectie Gezondheidszorg en Jeugd* (Health and Youth Care Inspectorate) |
| i.m., IM | Intramuscular |
| IMP | Investigational medicinal product |
| i.n., IN | Intranasal |
| INN | International nonproprietary name |
| irAE | Immune-related adverse event |
| IT | Information technology |
| IU | International unit(s) |
| i.v., IV | Intravenous |
| KHK | *Koronare Herzkrankheit* (coronary heart disease) |
| LDL | Low-density lipoprotein |
| MedDRA | Medical Dictionary for Regulatory Activities |
| µg | Microgram |
| mg | Milligram |
| MH | Medical history |
| MHH | *Medizinische Hochschule Hannover* (Hannover Medical School) |
| mL | Milliliter |
| MM | Medical monitor |
| n.a., NA | Not applicable |
| n.d., ND | Not determined |
| no. | Number |
| NSAID | Non-steroidal anti-inflammatory drug |
| OTC drugs | Over-the-counter drugs (i.e., non-prescription medicines) |
| PAOD | Peripheral arterial occlusive disease |
| pAVK | *Periphere arterielle Verschlusskrankheit* (peripheral arterial occlusive disease) |
| PCI | Percutaneous coronary intervention |
| PD-1 | Programmed cell death protein 1 |
| PD-L1 | Programmed cell death ligand 1 |
| PEI | *Paul-Ehrlich-Institut* (Federal Institute for Vaccines and Biomedicines) |
| p.i., PI | *Per inhalationem*, inhalative |
| PI | Principal investigator |
| p.o., PO | *Per os*, orally |
| p.r., PR | *Per rectum*, rectally |
| QM | Quality management |
| ® | Registered trademark |
| SAE | Serious adverse event |
| SAR | Serious adverse reaction |
| s.c., SC | Subcutaneous |
| s.l., SL | *Sub lingua*, sublingually |
| SmPC | Summary of product characteristics |
| SOP | Standard operating procedure |
| SPEAC | Safety Platform for Emergency vACcines |
| SUE | *Schwerwiegendes unerwünschtes Ereignis* (serious adverse event) |
| SUSAR | Suspected unexpected serious adverse reaction |
| t.d., TD | Transdermal |
| top., TOP | Topical |
| TTE | Transthoracic echocardiogram |
| TTS | Transdermal therapeutic system |
| U | Unit(s) |
| UE | *Unerwünschtes Ereignis* (adverse event) |
| Un, UN, Unk, UNK | Unknown |
| VEGF | Vascular endothelial growth factor |
| VWS | *Ministerie van Volksgezondheid, Welzijn en Sport* (Ministry of Health, Welfare and Sport) |
| x | Time(s) |
| XY | Dummy initials |
| XYZ | Dummy variable |

# Document history

| **Version number** | **Reason for revision** | **Effective date** |
| --- | --- | --- |
| 1.0 | Implementation of the initial version of the *Students’ Guide to Documentation in Clinical Trials* | 21 June 2022 |

# Introduction

From 31 January 2022 onwards, clinical trials in the European Union (EU) are conducted according to the Clinical Trials Regulation (Regulation (EU) No. 536/2014), which harmonizes the assessment and supervision processes for clinical trials throughout the EU. The Regulation will also apply to trials authorized under the previous legislation (i.e., EU Clinical Trials Directive (EC) No. 2001/20/EC) if these trials are still ongoing three years after the Clinical Trials Regulation has come into effect [1, 2].

Documentation in clinical trials adheres to the principles of Good Clinical Practice (GCP) as set forth by the International Council for Harmonisation of Technical Requirements for Registration of Pharmaceuticals for Human Use (ICH) Guideline for good clinical practice E6(R2). The ICH Guideline for good clinical practice E6(R2) defines GCP as *“an international ethical and scientific quality standard for designing, conducting, recording and reporting trials that involve the participation of human subjects. Compliance with this standard provides public assurance that the rights, safety and well-being of trial subjects are protected, consistent with the principles that have their origin in the Declaration of Helsinki, and that the clinical trial data are credible.”* [3]

The present guide is primarily addressed at students of human medicine and related disciplines who conduct documentation in clinical trials. However, other healthcare professionals working in clinical trials may also find the information contained in this guide relevant and useful. Of note, *“each individual involved in conducting a trial should be qualified by education, training, and experience to perform his or her respective task(s).”* [3]

After a brief description of the different roles in clinical trials (chapter 3) and an introduction into the principles of GCP (chapter 4), we are going to discuss the documentation of adverse events (chapter 5), concomitant medication (chapter 6), medical history (chapter 7), and quality control (chapter 8). The guide concludes with a concise medical dictionary German–English–Dutch (chapter 9) and with a list of references for further reading (chapter 10).

# Roles in clinical trials

In clinical trials we encounter different roles, the most important of which are outlined in **Table 1**.

Table 1: Description of roles in clinical trials

| **Role** | **Description** |
| --- | --- |
| Sponsor | An individual, company, institution, or organization which takes responsibility for the initiation, for the management, and for setting up the financing of a clinical trial. |
| Investigator, Principal Investigator | A person responsible for the conduct of the clinical trial at a trial site. If a trial is conducted by a team of individuals at a trial site, the investigator is the responsible leader of the team and may be called the principal investigator (PI). |
| Subinvestigator | An individual member of the clinical trial team designated and supervised by the (principal) investigator at a trial site to perform critical trial-related procedures and/or to make important trial-related decisions. |
| Subject; trial subject; study participant | An individual who participates in a clinical trial, either as a recipient of the investigational (medicinal) product or as a control. |
| Study Nurse; Research Nurse | A professional member of a clinical trial team who provides direct care and treatment for study participants, and who ensures that the trial subjects’ rights, safety, and well-being are protected throughout the study. |
| Investigational (medicinal) product | A pharmaceutical form of an active ingredient or placebo being tested or used as a reference in a clinical trial. |
| Monitor | A person appointed by the sponsor who oversees the progress of a clinical trial, and who ensures that the clinical trial is conducted, recorded, and reported in accordance with the protocol, SOPs, GCP, and regulatory requirements. |
| Auditor | A person who conducts systematic and independent examinations of trial-related activities and documents on behalf of the sponsor to determine whether trial-related activities were conducted, and the data were recorded, analyzed, and accurately reported according to the protocol, SOPs, GCP, and the applicable regulatory requirements (see also chapter 8.1.3.6). An auditor must be qualified by training and experience to conduct audits properly, and he or she must be independent of the clinical trial. |
| Inspector | A person who conducts official reviews of documents, facilities, records, and any other resources related to a clinical trial on behalf of a regulatory authority (see also chapter 8.1.3.7). |
| National Competent Authority | A medicines regulatory agency in a EU member state which reviews submitted clinical data, conducts inspections, and is responsible for the authorization of medicines that do not pass through the centralized procedure. National competent authorities (NCAs) in Germany are the Federal Institute for Drugs and Medical Devices (*Bundesinstitut für Arzneimittel und Medizinprodukte*) and the Federal Institute for Vaccines and Biomedicines (*Paul-Ehrlich-Institut*). In the Netherlands, NCAs are the Medicines Evaluation Board (*College ter Beoordeling van Geneesmiddelen*) and the Healthcare and Youth Care Inspectorate (*Inspectie Gezondheidszorg en Jeugd*) of the Ministry of Health, Welfare and Sport (*Ministerie van Volksgezondheid, Welzijn en Sport*). |
| Independent Ethics Committee | An independent body (a review board or a committee, institutional, regional, national, or supranational), constituted of medical professionals and non-medical members, whose responsibility it is to ensure the protection of the rights, safety, and well-being of human subjects involved in a trial and to provide public assurance of that protection. |

Role descriptions are adopted and modified from references [2], [3], and [4]. EU denotes European Union, GCP Good Clinical Practice, NCA national competent authority, PI principal investigator, and SOP standard operating procedure.

# General considerations and principles of Good Clinical Practice

## Writing utensils and text color

Handwritten documentation on case report forms (CRFs) (e.g., worksheets) should be performed with a biro, pen, or fineliner in document-proof (i.e., non-erasable) blue ink to ensure that the original can be distinguished from copies. Pencils, by contrast, are not acceptable as writing utensils as they are not document-proof. All handwritten entries on CRFs should be legible.

## Date formatting

Unless indicated otherwise, the date format is day–month–year:

- dd/mm/yyyy (e.g., 07/04/2021) or
- dd/mmm/yyyy (e.g., 07-Apr-2021).

Months should preferably be written in letters instead of numbers in order to prevent confusion with days: “07/04/2021” may be mistaken for 04 July 2021, whereas 07-Apr-2021 is unambiguous. As a rule, leading zeros should be included in the date format (e.g., 07-Apr-2021, not 7-Apr-2021) and years should be written out (e.g., 07-Apr-2021, not 07-Apr-21).

Examples of correct and incorrect date formatting are shown in **Table 2**.

Table 2: Date formatting

| **Correct date formatting** | **Incorrect date formatting** | **Reason(s) for incorrect date formatting** |
| --- | --- | --- |
| 03 November 2021 | 3 November 2021 | Omission of leading zero (“3” instead of “03”) |
| November 03, 2021 | November 3, 2021 | Omission of leading zero (“3” instead of “03”) |
| 03-Nov-2021 | 3.11.2021 | Omission of leading zero (“3” instead of “03”) |
| 03NOV2021 | 03.11.21 | Year incomplete (“21” instead of “2021”) |
| 03.11.2021 | 3.11.21 | Omission of leading zero(“3” instead of “03”) and year incomplete(“21” instead of “2021”) |
| 03/05/2021 | 3/5/2021 | Omission of leading zeros (“3” instead of “03” and “5” instead of “05”) |

## Handling of empty fields

In general, empty fields should be avoided as they leave room for (mis)interpretation: it remains unclear whether an empty field has simply been forgotten to be filled out or whether it was intentionally left blank, for example due to missing data. Empty fields should be crossed out or should be populated with “Unknown”/“Unk”/“UNK”/“Un”/“UN”, “Not applicable”/“NA”/“n.a.”, or “Not deter­mined”/“ND”/“n.d.”, depending on the context.

## Changes and corrections

All changes and corrections on CRFs must be *“dated, initialed, and explained (if necessary) and should not obscure the original entry”* [3], meaning that the original entry should still be identifiable after a change/correction has been made.

If longer text passages need to be crossed out manually, the use of a ruler is preferred to freehand line drawing. Please also make sure that an entry that needs to be corrected is crossed out entirely (i.e., over its full length; see also **Fig. 1** and **Fig. 2**).

## Worksheet check

The following points and issues should be checked and completed if necessary before a worksheet is finalized:

- The identification number (ID) of the clinical trial (usually the EudraCT number) must be included on the worksheet.
- The ID of the study site must be included on the worksheet.
- The participant/patient ID must be included on the worksheet.
- All empty fields must be populated as described in chapter 4.3.
- The page number (usually “page x of n”; see also chapter 8.2.4) must be included on the worksheet and must be consistent with previous and/or following associated worksheets.
- All entries on the worksheet must be dated and signed/initialed. The worksheet’s main date and signature represent exceptions to this rule as they can be left open until the worksheet is finalized (i.e., until all other fields have been populated). Thus, the worksheet’s main date and signature represent the most recent entries on the worksheet and serve as confirmation that the worksheet is finalized.

# Adverse events

## Definition of an adverse event

An adverse event (AE) is defined as *“any untoward medical occurrence in a patient or clinical investigation subject administered a pharmaceutical product and which does not necessarily have a causal relationship with this treatment. An AE can therefore be any unfavourable and unintended sign (including an abnormal laboratory finding), symptom, or disease temporally associated with the use of a medicinal (investigational) product, whether or not related to the medicinal (investigational) product.”* [3]

A central aspect of this AE definition is that a causal relationship between an investigational medicinal product (IMP) and a symptom is not necessary for this symptom to be considered (and documented as) an AE. A symptom must, however, be temporally associated with the administration of an IMP in order to be considered (and documented as) AE. A temporal association is given if a symptom occurs after or at IMP administration (and not before).

It is, however, advisable to discuss with the sponsor if the interpretation of the AE definition outlined above corresponds to the sponsor’s view. The sponsor may favor the opinion that all untoward medical occurrences after the screening visit constitute AEs, regardless of whether the IMP has already been administered or not. The result of this discussion should be documented and forwarded to all members of the study team so as to ensure consistent AE documentation.

In the following, some fictional examples are presented in order to illustrate what does and what does not constitute an AE in a clinical trial:

- One month after IMP administration, a study participant loses his job as a bank accountant because his employer is forced to lay off a considerable amount of the workforce amidst a financial crisis. This certainly is an untoward occurrence to the study participant, but not an untoward medical occurrence, and therefore does not constitute an AE.
- Six weeks after IMP administration, a study participant visits her general practitioner for a routine check-up examination. A venous blood sample is drawn and an elevated LDL (low-density lipoprotein) cholesterol value is detected for the first time. This can be regarded as an abnormal laboratory finding and, if considered clinically significant by the investigator, constitutes an AE. An appropriate AE documentation in this example would be “LDL hypercholesterolemia”.
- At the screening and enrollment visit at the study center, a study participant cuts his left thumb on a sharp paper edge while browsing through the informed consent form (ICF). The study participant has not signed the ICF yet and, consequently, the IMP has not been administered yet. A tiny bleeding occurs which is treated with disinfection, local compression, and the application of a plaster. This injury does not constitute an AE because the IMP has not been administered yet. The injury could, however, be listed in the study participant’s medical history, but since in this case the trauma is minute, documentation is not mandatory.
- At the screening and enrollment visit, a study participant states that she plans to undergo an elective hip replacement surgery in three months which will be associated with a hospital stay and ensuing rehabilitation. The investigator diligently evaluates the inclusion and exclusion criteria of the clinical trial and comes to the conclusion that participation in the clinical trial is possible. The planned elective hip replacement surgery is documented in the participant’s study file and, once completed, does not constitute an AE provided that no complications arise during the surgery or associated hospital stay.
- Two weeks after IMP administration a study participant dies as passenger in an airplane crash. Even though a causal relationship between IMP administration and the airplane crash is not reasonably possible, the event nevertheless constitutes an AE. Since the event is fatal, it even represents a *serious* AE, as we shall discuss in detail in chapter 5.2.

## Definition of a serious adverse event

A serious adverse event (SAE) is an AE that:

- results in death/is fatal,
- is life-threatening,
- requires inpatient hospitalization or prolongation of existing hospitalization,
- results in persistent or significant disability/incapacity, or
- is a congenital anomaly/birth defect [3].

In addition, the Detailed guidance on the collection, verification and presentation of adverse event/reaction reports arising from clinical trials on medicinal products for human use (CT‑3) specifies that *“some medical events may jeopardise the subject or may require an intervention to prevent one of the above characteristics/consequences. Such events […] should also be considered as ‘serious’ in accordance with the definition. Medical and scientific judgement should be exercised in deciding whether an event is ‘serious’ in accordance with these criteria.”* [5] An example might be an extremely severe migraine attack which requires administration of zolmitriptan nasal spray by the participant to prevent significant incapacity. According to CT‑3, an event like this migraine attack should also be considered as serious [5], even though none of the five formal seriousness criteria outlined above are fulfilled.

If you have the suspicion (and even more so if you are certain) that an AE reported by a study participant fulfills at least one of the seriousness criteria, please inform one of the (sub)investigators without delay, as all SAEs must be reported immediately to the sponsor by the (sub)investigator.

Of note, an unplanned hospital visit by a study participant without subsequent overnight hospitalization (e.g., a visit to the emergency department with surgical wound closure of a laceration and subsequent discharge of the study participant into the ambulatory setting) constitutes an AE, but not an SAE.

## Seriousness versus severity

For the documentation of AEs it is essential to differentiate between the terms *seriousness* and *severity*. An AE is *serious* (and hence becomes an SAE) if at least one of the seriousness criteria outlined in chapter 5.2 is fulfilled (or if the (sub)investigator considers the AE as *serious* according to CT‑3 [5]).

*Severity*, on the other hand, describes the clinical intensity of an AE. In its simplest form, severity is graded as “mild”, “moderate”, or “severe”. Of note, a *severe* AE is not necessarily *serious*, and—vice versa—an SAE is not necessarily *severe*.

## Common Terminology Criteria for Adverse Events

The National Cancer Institute of the United States Department of Health and Human Services developed the Common Terminology Criteria for Adverse Events (CTCAE), which are widely used in clinical trials for AE reporting. The CTCAE are a descriptive tool that provides a grading (severity) scale for each AE [6].

The CTCAE are based on the Medical Dictionary for Regulatory Activities (MedDRA), an extensive and highly specific standardized medical terminology which was promulgated by the ICH in the late 1990s [7]. The CTCAE severity scale encompasses grades 1 to 5, with higher grades indicating an increasing severity, as delineated in **Table 3**. Of note, the descriptors of CTCAE grades 3 and 4 show a substantial overlap with the SAE criteria (chapter 5.2); CTCAE grade 5 is always an SAE.

In addition, CTCAE grades can be a practical aid in assessing the clinical significance of laboratory abnormalities in a standardized manner.

Table 3: Common Terminology Criteria for Adverse Events severity grading

| **Grade** | **Description** |
| --- | --- |
| **1** | **Mild**; asymptomatic or mild symptoms; clinical or diagnostic observations only; intervention not indicated |
| **2** | **Moderate**; minimal, local or noninvasive intervention indicated; limiting age-appropriate instrumental ADL (e.g., preparing meals, shopping for groceries or clothes, using the telephone, managing money, etc.) |
| **3** | **Severe or medically significant** but not immediately life-threatening; hospitalization or prolongation of hospitalization indicated; disabling; limiting self-care ADL (e.g., bathing, dressing and undressing, feeding self, using the toilet, taking medications, and not bedridden) |
| **4** | **Life-threatening** consequences; urgent intervention indicated |
| **5** | **Death** related to AE |

A semi-colon denotes “or” within the description of the CTCAE grades. Underlined terms highlight overlap with SAE criteria (chapter 5.2). ADL denotes activities of daily living, AE adverse event, CTCAE Common Terminology Criteria for Adverse Events, and SAE serious adverse event. The table is adopted and modified from reference [6].

## Definition of an adverse event of special interest

An adverse event of special interest (AESI) is defined as an AE (serious or nonserious) which is of particular scientific or medical concern to the sponsor, and which is specified in the clinical trial protocol. In case of occurrence of an AESI, continuous monitoring and rapid communication of the investigator with the sponsor is required and might need further investigation in order to characterize and fully understand the event. Depending on the nature of the AESI, rapid notification and communication by the trial sponsor to other parties (e.g., regulators) might also be necessary [8, 9]. Two types of AESIs that have recently aroused significant interest and that have gained particular relevance in clinical trials are briefly described subsequently.

### Coronavirus disease 2019-related adverse events of special interest

The Brighton Collaboration is an international consortium which was established in 1999 to develop definitions and guidelines for potential adverse events following immunization [10]. Since 2019, the Brighton Collaboration has been cooperating with the Coalition for Epidemic Preparedness Innovation (CEPI) for the development of tools within the Safety Platform for Emergency vACcines (SPEAC) in order to guide scientific developers, regulatory agencies, and health authorities through safety evaluations of vaccines with new technologies. The collaborating institutions have substantially increased their activities since the beginning of the coronavirus disease 2019 (COVID‑19) pandemic to facilitate and safeguard the assessment of the safety of COVID‑19 vaccines [11]. The SPEAC project has generated a comprehensive list of AESIs for safety monitoring to harmonize safety assessment of CEPI-funded vaccines [12]. The list is updated quarterly. Examples of COVID‑19-related AESIs include subacute thyroiditis, pancreatitis, and rhabdomyolysis [13].

### Immune-related adverse events

The advent of immune checkpoint inhibition as therapeutic strategy has revolutionized oncology and has vastly expanded the antineoplastic armamentarium. Monoclonal antibodies approved for oncological indications target immune checkpoints CTLA‑4 (cytotoxic T‑lymphocyte antigen 4; e.g., ipilimumab), PD‑1 (programmed cell death protein 1; e.g., pembrolizumab), as well as its ligand PD‑L1 (e.g., atezolizumab). These potent antineoplastic agents are clinically used against a plethora of malignancies such as skin, bladder, breast, liver, kidney, lung, and head and neck cancer, among others [14]. However, interference with the regulation of the immune system is accompanied by considerable side effects termed immune-related adverse events (irAEs), which result from off-target immune responses against noncancerous tissues. Immune-related adverse events can affect virtually any organ of the body; important examples of irAEs include—but are not limited to—dermatitis, thyroiditis, hypophysitis, hepatitis, nephritis, colitis, myocarditis, and pneumonitis [15, 16].

## Causality assessment

A critical step in the evaluation of (serious) AEs is the assessment of their relatedness to the IMP. This causality assessment requires extensive medical and scientific judgment and is therefore performed exclusively and independently from each other by both the (sub)investigator and the sponsor. If either (or both) considers (consider) the causal relationship between the (S)AE and the IMP at least possible, the (S)AE becomes a (serious) adverse reaction ((S)AR).

## Definition of a suspected unexpected serious adverse reaction

A suspected unexpected serious adverse reaction (SUSAR) is defined as an AE that is serious (chapter 5.2), unexpected, and that is being assessed as related to IMP administration (the tested IMP or comparators). “Unexpected” in this context means that the nature or severity of the event are not consistent with the applicable reference document (i.e., the investigator’s brochure (IB) for an unapproved IMP [17] or the summary of product characteristics (SmPC) for an approved product). Of note, the decision whether or not an event constitutes a SUSAR is made by the sponsor, not by the (sub)investigator.

## Documentation of adverse events

### Principle of parsimony

As a rule, AE documentation in clinical trials should be performed parsimoniously, meaning that overarching diagnoses should be preferred to single symptoms. For instance, “rhinorrhea”, “cough”, and “tiredness” should not be documented as separate events if an overarching diagnosis, such as “common cold”, comprises and sufficiently explains all of these symptoms (**Fig. 1**).


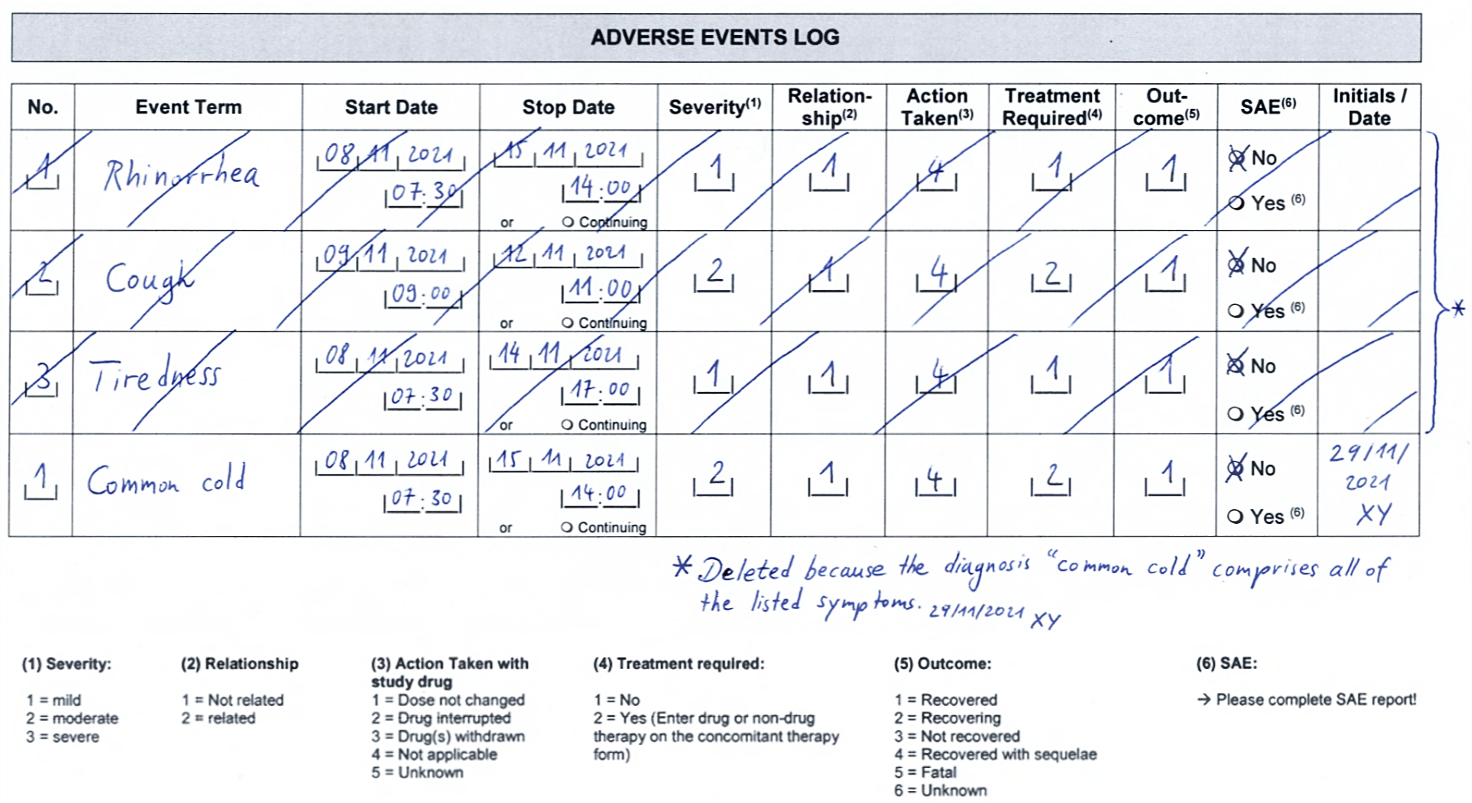


Fig. 1: Use of overarching diagnoses in lieu of single symptoms

Initially, the events “rhinorrhea”, “cough”, and “tiredness” were listed as separate events (AEs no. 1–3). During AE review it became clear that these three events were related to an overarching diagnosis (i.e., “common cold”). Hence, AEs no. 1–3 were deleted (crossed out) and “common cold” was stated as the new AE no. 1. Of note, the overarching diagnosis “common cold” adopts the longest time span (07:30 on 08-Nov-2021 to 14:00 on 15-Nov-2021) as well as the highest severity score (“2”; i.e., moderate) of the underlying three symptoms. Treatment was required for the event “common cold” because the underlying symptom “cough” was treated with codeine.

AE denotes adverse event, XY dummy initials.

### Examples of adverse event documentation

In the following, the GCP-conform documentation of AEs is exemplified and discussed from a practical viewpoint. Special emphasis is put on how to update the AE log (**Fig. 2**) and on how to document SAEs and accidents **Fig. 3**).


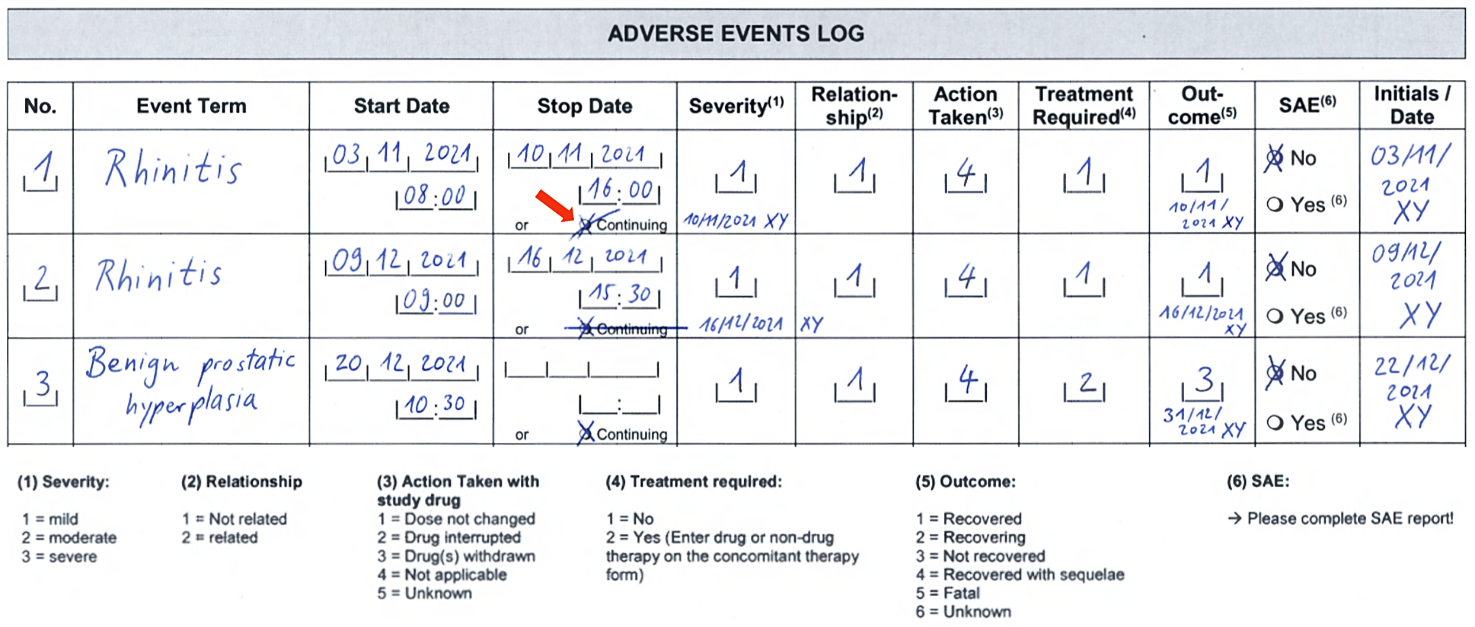


Fig. 2: Updating the adverse events log

**AE no. 1**: The event “Rhinitis” started on 03-Nov-2021 and was documented in the AE log on the same day. The stop date was left open during documentation on 03-Nov-2021 and the box “Continuing” was checked. The event was finished on 10-Nov-2021 and the outcome “Recovered” was entered into the corresponding field on the same day. The box “Continuing”, however, was not properly crossed out (i.e., not over the entire length of the box and word; red arrow) so that the implemented change may easily be overlooked. This problem was more adequately solved in AE no. 2.

**AE no. 2** describes a similar scenario as AE no. 1. This time, however, the box “Continuing” was adequately crossed out once the event was finished, taking advantage of a ruler. This documentation style should be preferred.

**AE no. 3**: The event “Benign prostatic hyperplasia” was detected during a visit of the study participant at his urologist on 20-Dec-2021. During AE documentation two days later (on 22-Dec-2021), the box “Continuing” was checked because the event is permanent. As a therapy with tamsulosin was initiated by the urologist, the field “Treatment required” was populated with a “2” (i.e.; “Yes”). At the end of the clinical trial on 31-Dec-2021, the outcome “Not recovered” was entered into the corresponding field since the event was still ongoing at this time point.

AE denotes adverse event, XY dummy initials.


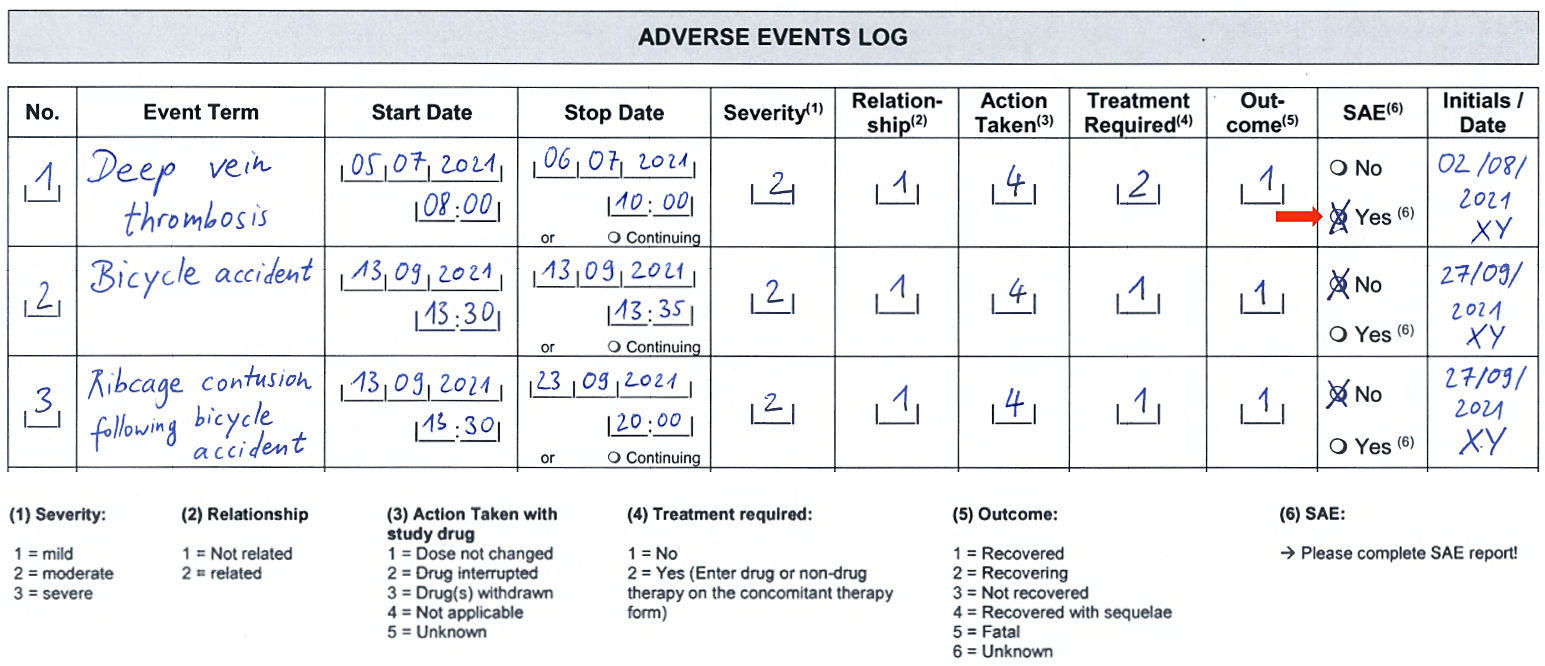


Fig. 3: Documentation of a serious adverse event and documentation of an accident with ensuing injury

**AE no. 1**: The study participant developed a deep vein thrombosis during a long-distance flight to Australia on 05-Jul-2021. The diagnosis was established in an Australian hospital on the same day, and the study participant was hospitalized for one night to exclude pulmonary embolism. The event was rated as *serious* (red arrow) because the SAE criterion “hospitalization” was fulfilled. A treatment with rivaroxaban was initiated and the patient was discharged on 06-Jul-2021 as recovered. The severity of the event was rated as moderate since the patient could be released from hospital already after one day when pulmonary embolism had been excluded and oral anticoagulation had been instituted. As the study participant was on vacation when the event happened, the study center was informed with delay on 02-Aug-2021. Notwithstanding, once the study center had become aware of the seriousness of the event, an SAE report was submitted to the sponsor immediately on the same day (i.e., 02-Aug-2021).

**AEs no. 2 and no. 3**: The study participant had a bicycle accident on 13-Sep-2021, which led to a ribcage contusion. Both the accident and the ensuing injury were (correctly) documented as separate events.

AE denotes adverse event, SAE serious adverse event, and XY dummy initials.

# Concomitant medication

The concomitant medication (ConMed) comprises all the drugs a study participant is taking regularly and on demand (“as needed”, *pro re nata*), including non-prescription medicines (“over-the-counter” (OTC) drugs). Non-prescription medicines are described in detail in chapter 6.5. Common examples of prescription medicines are antihypertensives, antidiabetics, lipid-lowering agents, platelet aggregation inhibitors, anticoagulants, non-steroidal anti-inflammatory drugs (NSAIDs), opioid analgesics, and antidepressants. In its simplest form, the ConMed log includes drugs which the study participant is already taking at screening/enrollment as well as drugs that are newly added to his or her medication regimen during the course of the clinical trial (e.g., drugs for AE treatment or vaccinations). More refined variants of the ConMed log are also possible, for example, the differentiation into “Previous ConMed Log”, “Study Disease Medication”, or “General Medication”.

In this chapter we are going to introduce medication terminology (chapter 6.1), posology (chapter 6.2), the different routes of drug administration (chapter 6.3), application frequencies (chapter 6.4), and we are also going to highlight particularities regarding the documentation of non-prescription medicines (chapter 6.5). We conclude this chapter with the discussion of three fictional ConMed scenarios (chapter 6.6).

## Concomitant medication terminology

As a rule, generic names (i.e., international nonproprietary names (INN); e.g., rivaroxaban) should be preferred to brand names (e.g., Xarelto®). If in exceptional cases a drug’s brand name is used, its corresponding generic name should be added in parentheses (e.g., “Xarelto® (rivaroxaban)”). **Table 4** summarizes recommendations regarding ConMed terminology.

Table 4: Concomitant medication terminology

| **Correct ConMed terminology** | **Incorrect ConMed terminology** | **Reason(s) for incorrect ConMed terminology** |
| --- | --- | --- |
| Acetylcysteine | ACC | Use of an unexplained abbreviation |
| Thomapyrin® CLASSIC (acetylsalicylic acid + paracetamol + caffeine) | Thomapyrin | As the brand name “Thomapyrin” comprises three different combination products (i.e., Thomapyrin® CLASSIC (250 mg acetylsalicylic acid + 200 mg paracetamol + 50 mg caffeine), Thomapyrin® INTENSIV (250 mg acetylsalicylic acid + 250 mg paracetamol + 50 mg caffeine), and Thomapyrin® TENSION DUO (400 mg ibuprofen + 100 mg caffeine)), it remains unclear which of these combination products the entry is referring to.  Besides, the “®” sign (registered trademark) has been omitted. |
| Simvastatin | Simva | Medical jargon used instead of INN |

ConMed denotes concomitant medication, INN international nonproprietary name, and ® registered trademark.

## Posology

### Leading and trailing zeros

As a rule, leading zeros should be included in medication dosages, whereas trailing zeros should be omitted (**Table 5**).

Table 5: Leading and trailing zeros in medication dosages

| **Correct medication dosage** | **Incorrect medication dosage** | **Reason for incorrect medication dosage** |
| --- | --- | --- |
| Digitoxin 0.1 mg | Digitoxin .1 mg | Omission of leading zero (depending on the legibility of the handwriting, confusion with digitoxin 1 mg is possible) |
| Clonidine 0.3 mg | Clonidine .3 mg | Omission of leading zero (depending on the legibility of the handwriting, confusion with clonidine 3 mg is possible) |
| Lorazepam 0.5 mg | Lorazepam .5 mg | Omission of leading zero (depending on the legibility of the handwriting, confusion with lorazepam 5 mg is possible) |
| Lorazepam 1 mg | Lorazepam 1.0 mg | Use of trailing zero (depending on the legibility of the handwriting, confusion with lorazepam 10 mg is possible) |
| Bisoprolol 5 mg | Bisoprolol 5.0 mg | Use of trailing zero (depending on the legibility of the handwriting, confusion with bisoprolol 50 mg is possible) |
| Furosemide 20 mg | Furosemide 20.0 mg | Use of trailing zero (depending on the legibility of the handwriting, confusion with furosemide 200 mg is possible) |

### Dosages in micrograms

When you document dosages in micrograms (e.g., “levothyroxine 75 µg”, please make sure to write the Greek letter “µ” very clearly so as to avoid confusion with the Latin letter “m”, as confusion of µg with mg will result in a dose that is 1000 times higher than intended. Alternatively, you may use the abbreviations mcg and mg to denote micrograms and milligrams, respectively.

## Routes of administration

Adopting a simplified model, the different routes of drug administration can be differentiated into enteral and parenteral. Enteral administration is achieved through the gastrointestinal tract and includes oral, sublingual, buccal, and rectal administration. Parenteral administration, on the other hand, is any route that is not enteral. There is a plethora of parenteral routes of administration such as intravenous, intramuscular, subcutaneous, intradermal, inhalative, intranasal, topical, transdermal, intralesional, ophthalmic/ocular, intravitreal, auricular/otic, vaginal, and intrathecal administration [18].

In the following, the enteral (chapter 6.3.1) and the parenteral routes of administration (chapter 6.3.2) are described and common examples of drugs applied via these routes are presented. Please note that neither the listed routes of administration nor the medication examples are exhaustive.

### Enteral administration

#### Oral (*per os*, p.o., PO)

Oral administration is a convenient and most commonly used drug administration route. The primary site of drug absorption is the small intestine. Before reaching the systemic circulation, a significant proportion of orally administered drugs is metabolized and eliminated by the intestine and, more importantly, the liver (first-pass effect). Examples of orally administered drugs include, but are not limited to, analgesics, antihypertensives, platelet aggregation inhibitors and oral anticoagulants, immunosuppressants and immune modulators, antiparkinson agents, antidepressants, antipsychotics, and so forth. Units of orally applied drugs are g, mg, and µg (the latter especially for levothyroxine).

#### Sublingual (s.l., SL)

By applying a drug directly under the tongue (i.e., sublingually), the disadvantage of the first-pass effect encountered upon oral drug administration can be circumvented as the capillaries of the sublingual mucosa drain into the superior vena cava without involvement of the hepatic portal vein system. Sublingually applied drugs are rapidly absorbed because the sublingual mucosa is highly permeable and vascularized. Hence, if rapid onset of pharmacological action is therapeutically desired, the sublingual route of drug administration offers advantages. Besides, drugs that display a high first-pass effect (and that would be degraded by the liver to a large extent before reaching systemic circulation if applied orally) can be administered sublingually. Common examples of sublingually administered drugs include, among others, nitroglycerin, lorazepam, and buprenorphine.

#### Buccal

Similar to the sublingual route (chapter 6.3.1.2), buccal drug administration offers the advantage of bypassing the first-pass effect. The drug is placed between the gingiva and the inner lining of the cheek. In comparison with sublingual mucosa, buccal tissue is less permeable which results in a slower absorption rate. Drugs that are available in buccal forms are, for example, asenapine, buprenorphine, fentanyl, and midazolam.

#### Rectal (*per rectum*, p.r., PR)

The rectal mucosa is highly vascularized and absorbs medications rapidly and effectively. Similar to the sublingual (chapter 6.3.1.2) and buccal routes (chapter 6.3.1.3), drugs delivered to the distal one-third of the rectum largely avoid the first-pass effect, allowing for greater bioavailability of many medications compared to the oral route. Rectal administration of drugs is achieved by the insertion of suppositories or rectal tubes/rectioles containing, for example, paracetamol, diazepam, or laxatives.

#### Other enteral routes of administration

Other enteral routes of drug administration are, for example, sublabial administration (placing a drug between the gingiva and the lip), administration directly into the stomach via nasogastric tube or gastrostomy, or administration directly into the small intestine via duodenal tube.

### Parenteral administration

#### Intravenous (i.v., IV)

Intravenous injection/infusion is the most common parenteral route of drug administration. Per definition, intravenously applied drugs have a bioavailability of 100 %. Examples of intravenously applied medications are, for example, chemotherapeutic agents, monoclonal antibodies, and drugs used during general anesthesia, such as propofol, opioid analgesics (fentanyl, remifentanil, morphine, hydromorphone, etc.), and muscle relaxants. Another example is insulin which can be administered intravenously in inpatient settings (e.g., on endocrinology wards or in intensive care units).

#### Intramuscular (i.m., IM)

Common sites for intramuscular injections are the deltoid and the gluteal muscle. As muscles possess larger and more numerous blood vessels than subcutaneous tissue, drug absorption following intramuscular injection is faster than after subcutaneous (chapter 6.3.2.3) or intradermal injection (chapter 6.3.2.4). The vast majority of vaccines are administered intramuscularly. Examples of intramuscularly applied drugs include glucocorticoids (e.g., triamcinolone acetonide), antibiotics, and long-acting injectable antipsychotics (e.g., risperidone, paliperidone, olanzapine, and haloperidol).

#### Subcutaneous (s.c., SC)

Subcutaneous injections are administered into the fatty tissue of the subcutis, the layer of skin located directly beneath the dermis and epidermis. Drug absorption following subcutaneous injection is slower than after intravenous (chapter 6.3.2.1) or intramuscular injection (chapter 6.3.2.2). Insulin and insulin analogs, low-molecular-weight heparins, interferon beta‑1b, methotrexate, and certain biologicals (e.g., etanercept, belimumab, dupilumab, ofatumumab, etc.) are examples of subcutaneously applied drugs. Of note, insulin and insulin analogs can be applied subcutaneously with different devices (pen, pump, etc.), which should be taken into consideration during documentation.

#### Intradermal (i.d., ID)

Intradermal administration is the injection of a substance into the dermis, which is located between the epidermis and the subcutis. This route of administration is rarely used in comparison with intramuscular (chapter 6.3.2.2) or subcutaneous injections (chapter 6.3.2.3). Intradermal injections are used, for example, for tuberculosis and allergy testing.

#### Inhalative (*per inhalationem*, p.i., PI)

Inhalation is the preferred route of administration for drugs that act specifically on the lungs for the treatment of diseases like asthma or chronic obstructive pulmonary disease (COPD). Examples of inhaled drugs are beta‑2 agonists (e.g., salbutamol, fenoterol, terbutaline, formoterol, salmeterol, olodaterol, vilanterol, etc.), muscarinic antagonists (e.g., tiotropium, ipratropium, glycopyrronium, etc.), and glucocorticoids (e.g., fluticasone, beclomethasone, budesonide, etc.). Inhaled medications are usually dosed in puffs (e.g., “2 puffs in the morning, 2 puffs in the evening”).

#### Intranasal (i.n., IN)

The intranasal route via nasal sprays can be used for local treatment of the nasal mucosa as well as to achieve systemic drug exposure. Glucocorticoids (e.g., mometasone furoate, budesonide), antihistamines (e.g., azelastine, levocabastine), and nasal decongestants (e.g., xylometazoline) are administered intranasally to achieve a local treatment effect. Sumatriptan (for migraine attacks), calcitonin (for osteoporosis), and nicotine (for smoking cessation), on the other hand, are applied intranasally to achieve systemic drug exposure and systemic pharmacological effects. The dosage of nasal sprays is usually documented in “applications”, “sprays”, or “puffs” (e.g., “1 application in each nostril up to 3 times daily”, “1 spray per nostril 2 times daily”, or “2 puffs per nostril per day”).

#### Topical (top., TOP)

In topical therapy, the skin itself and/or underlying anatomical structures are the target organ(s). Topically applied drugs are used to treat inflammatory skin disorders (e.g., psoriasis, atopic dermatitis) and skin infections (viral, bacterial, or fungal), among others. Examples of topically administered drugs include glucocorticoids (e.g., prednicarbate, mometasone furoate, clobetasol propionate, etc.), calcineurin inhibitors (tacrolimus, pimecrolimus), antifungals (terbinafine, clotrimazole, ciclopirox, etc.), antivirals (e.g., acyclovir), and NSAIDs (e.g., diclofenac). Topically administered drugs are applied as creams, ointments, gels, lotions, suspensions, pastes, or powders. Units for topical preparations are the strip length of a cream, ointment, etc. in cm or the “fingertip unit”, a semiquantitative measure representing the distance from an individual’s distal interphalangeal joint of his or her index finger to the fingertip (ca. 2–3 cm).

#### Transdermal (t.d., TD)

In contrast to topical therapy (chapter 6.3.2.7), the skin is not the target organ in transdermal therapy, but merely serves as the site of drug absorption into the circulation. Transdermal therapy is usually accomplished by the application of patches (transdermal therapeutic systems (TTS)), examples of which are nicotine (for smoking cessation), rivastigmine (for Alzheimer’s disease), rotigotine (for Parkinson’s disease), granisetron (for chemotherapy-induced nausea and vomiting), nitroglycerin (for angina pectoris), oxybutynine (for urinary incontinence), scopolamine (for motion sickness), buprenorphine, and fentanyl TTS (both for pain therapy) [19].

#### Intralesional

Certain skin disorders, such as acne inversa (hidradenitis suppurativa), can be treated by injections of drugs (e.g., triamcinolone acetonide) directly into skin lesions (i.e., intralesional drug administration).

#### Ophthalmic/ocular

Ophthalmic/ocular administration of drugs is accomplished by the use of eye drops, gels, and ointments. Examples of ophthalmically applied drugs include, among others, antiglaucoma agents (e.g., timolol, latanoprost, dorzolamide), antibiotics, mydriatics (e.g., phenylephrine, tropicamide), and miotics (e.g., pilocarpine).

#### Intravitreal

Intravitreal injection is the administration of drugs into the eye, directly into the vitreous humor. Intravitreal drug administration is used to treat eye diseases such as age-related macular degeneration, diabetic retinopathy, and infections located inside the eye. Examples of intravitreally applied drugs include, for example, antibiotics (e.g., vancomycin, ceftazidime, moxifloxacin), antimycotics (e.g., amphotericin B, voriconazole), antivirals (e.g., ganciclovir, foscarnet, cidofovir), anti-VEGF agents (e.g., bevacizumab, ranibizumab, aflibercept), and glucocorticoids (e.g., triamcinolone acetonide, dexamethasone).

#### Auricular/otic

Certain drugs, such as antibiotics (e.g., ciprofloxacin), glucocorticoids (e.g., hydrocortisone), or local anesthetics (e.g., benzocaine), can be instilled as ear drops (solutions, suspensions) into the auditory canal to treat infections, inflammations, or to achieve local anesthesia.

#### Vaginal

Certain drugs, such as estrogens, antibiotics, and antimycotics, may be administered vaginally as a cream, gel, solution, tablet, suppository, or ring.

#### Int**r**athecal

Intrathecal administration is the injection of drugs into the spinal canal so that they reach the cerebrospinal fluid. Intrathecal drug administration may be used for spinal anesthesia, chemotherapy (e.g., methotrexate, cytarabine), pain therapy (e.g., opioid analgesics, ziconotide), or the treatment of spastic cerebral palsy (e.g., baclofen). Of note, the rare neuromuscular disorder spinal muscular atrophy can be treated by intrathecal administration of nusinersen.

#### Other parenteral routes of administration

Other parenteral routes of drug administration are, for example, epidural/peridural (into the epidural space), intra-arterial (into an artery), intra-articular (into a joint), intracavernous (into the corpus cavernosum of the penis), intraosseous (into the bone marrow), intraperitoneal (into the peritoneal cavity), and intravesical (into the urinary bladder).

## Drug application frequencies

In order to achieve maximum therapeutic benefit and to avoid over- or underdosing, every drug has its preferred application interval. **Table 6** lists abbreviations for common drug application frequencies. The tabulated abbreviations are widely used in clinical practice and can be considered as medical standard. Nevertheless, it must be noted that abbreviations always bear a certain confusion potential; for example, the abbreviations QD (every day) and QID (four times a day) can easily be confounded. Therefore, depending on the context, it may be more prudent and advisable to use the corresponding colloquial English equivalents when documenting application frequencies. Thus, misinterpretations can be avoided.

Another alternative is the use of a notation scheme such as “ibuprofen 600 mg 1–1–1” or “lorazepam 0.5 mg 1–1–1–1”, describing that one 600‑mg tablet of ibuprofen is taken in the morning, at noon, and in the evening, whereas one 0.5‑mg tablet of lorazepam is taken in the morning, at noon, in the evening, and at night. One 2.5‑mg tablet of bisoprolol taken in the morning and in the evening can be denoted as “bisoprolol 2.5 mg 1–0–1”. Two 20‑mg tablets of furosemide taken in the morning and one 20‑mg tablet of furosemide taken at noon can be described as “furosemide 20 mg 2–1–0”.

Table 6: Abbreviations of drug application frequencies, their Latin origin, their meaning, and corresponding colloquial English equivalents

| **Abbreviation** | **Latin origin** | **Meaning and colloquial English equivalent(s)** |
| --- | --- | --- |
| q.d., qd, QD | *Quaque die* | Every day, (once) daily, 1x per day, 1x/d |
| b.i.d., bid, BID;  b.d.s., bds, BDS | *Bis in die*;  *bis die sumendum* | Two times a day, twice a day, twice daily, 2x per day, 2x/d |
| t.i.d., tid, TID;  t.d.s., tds, TDS | *Ter in die;*  *ter die sumendum* | Three times a day, 3x per day, 3x/d |
| q.i.d., qid, QID;  q.d.s., qds, QDS | *Quater in die*;  *quater die sumendum* | Four times a day, 4x per day, 4x/d |
| alt. d., alt. dieb.;  dieb. alt.;  EOD;  q.o.d., qod, QOD | *Alternis diebus*;  *diebus alternis*;  every other day;  *quaque altera die* | Every other day, on alternate days |
| q.wk., qw; QWK | — | Once a week, (once) weekly |
| q2w, Q2W | — | Every two weeks |
| q4w, Q4W | — | Every four weeks |
| p.r.n., PRN | *Pro re nata* | As needed, on demand |

d denotes day, w(k) week, and x time(s).

## Non-prescription medicines

Non-prescription medicines (OTC drugs) comprise phytopharmaceuticals, homeopathic prepa­rations, vitamins, minerals, and enzyme preparations, only to name a few. Phytopharmaceuticals often contain a large number of herbal ingredients. Therefore, and as an exception to the generally preferred use of generic names (chapter 6.1), it is advisable to list the brand name for phytopharmaceuticals instead of detailing all of the herbal ingredients separately.

When inquiring the indication for non-prescription medicines of study participants, experience shows that the precise medical reason for intake often remains elusive. Lay explanations for the intake of non-prescription medicines, such as “to feel better”, “to strengthen my immune system”, and so forth, should not be entered into the ConMed log literally. Acceptable alternatives are, for example, “prophylaxis of common cold”, “prophylaxis of vitamin XYZ deficiency”, “prophylaxis of mineral ABC deficiency”, etc., being fully aware that such “indications” are often pseudo-medical and not scientifically proven.

## Examples of concomitant medication documentation

In the following, three fictional ConMed logs are presented and explained in detail (**Fig. 4**, **Fig. 5**, and **Fig. 6**).


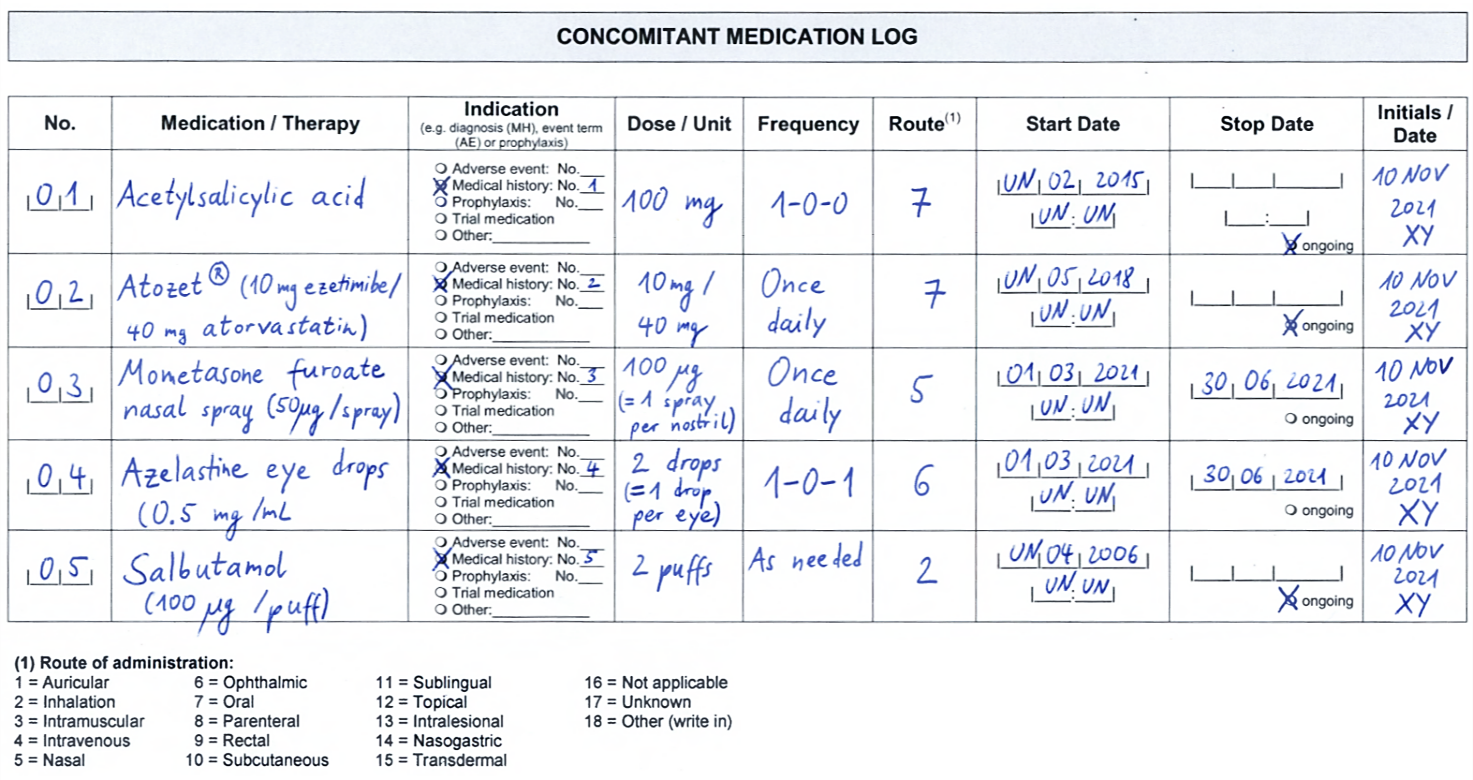


Fig. 4: Documentation of concomitant medication—first example

**ConMed no. 01**: Acetylsalicylic acid is being prescribed for coronary heart disease (= Medical history no. 1). Frequency “1–0–0” denotes application of acetylsalicylic acid once daily in the morning.

**ConMed no. 02**: Atozet® is a fixed-dose combination of ezetimibe and atorvastatin to treat hyper­cholesterolemia (= Medical history no. 2).

**ConMed no. 03**: Mometasone furoate nasal spray was used from 01-Mar-2021 to 30-Jun-2021 to treat seasonal allergic rhinitis (= Medical history no. 3).

**ConMed no. 04**: Azelastine eye drops were used from 01-Mar-2021 to 30-Jun-2021 to treat seasonal allergic conjunctivitis (= Medical history no. 4). Frequency “1–0–1” denotes application of azelastine eye drops twice daily (in the morning and in the evening). Please note that the right parenthesis (i.e., “)”) in “Azelastine eye drops (0.5 mg/mL” is missing. This is a minor error detected during ConMed review that can easily be rectified.

**ConMed no. 05**: Salbutamol is inhaled on an as-needed basis if the study participant is suffering from an acute attack of allergic asthma (= Medical history no. 5).

ConMed denotes concomitant medication, ® registered trademark, and XY dummy initials.


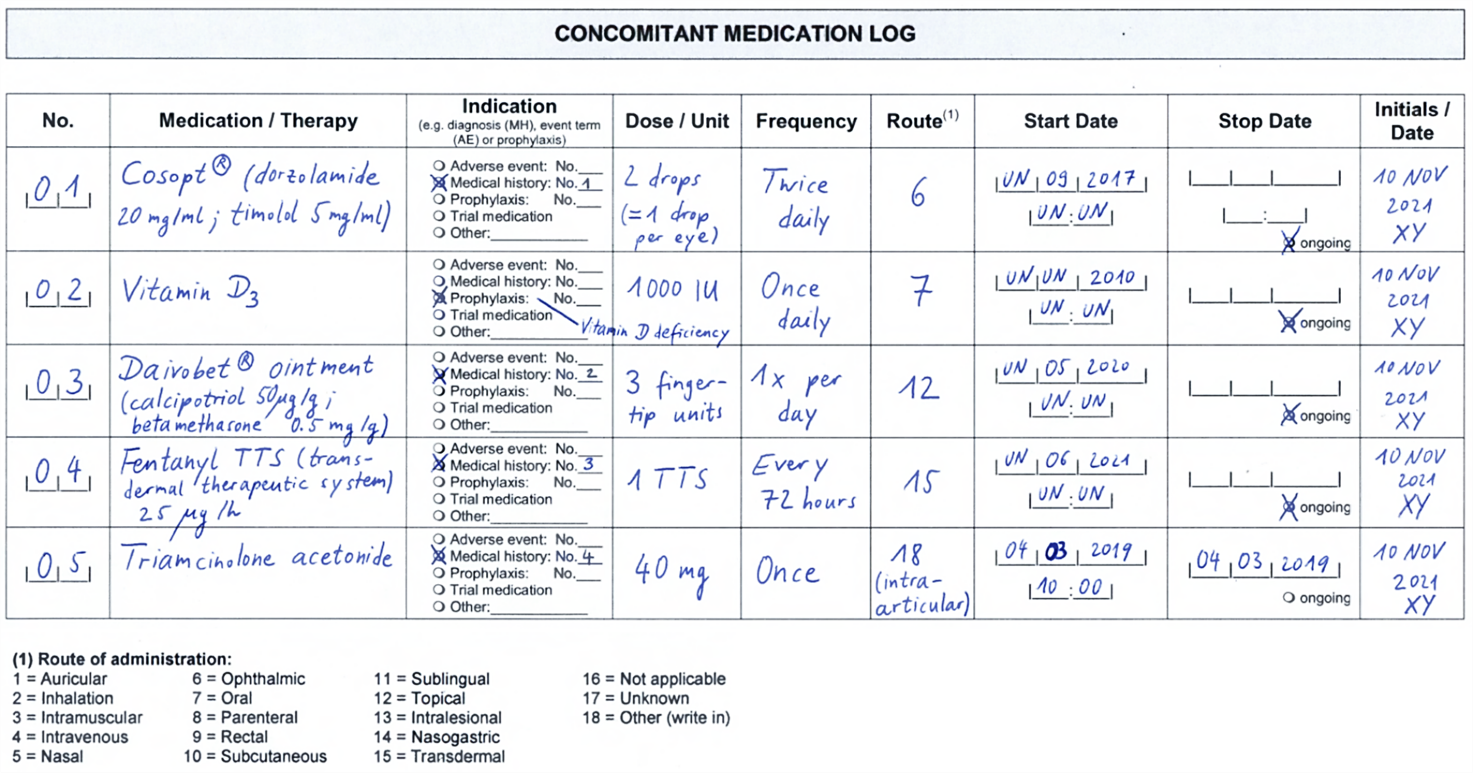


Fig. 5: Documentation of concomitant medication—second example

**ConMed no. 01**: Cosopt® eye drops (i.e., combination product of dorzolamide and timolol) are used to treat glaucoma (= Medical history no. 1). Two drops (i. e., one drop per eye) are administered twice daily, so that—taken together—the study participant applies 4 drops of Cosopt® per day.

**ConMed no. 02**: The study participant could not precisely state why she was taking vitamin D_3_ daily. For the purpose of documentation, “prophylaxis of vitamin D deficiency” is an acceptable indication if the (e)CRF does not allow the entry “Unknown”.

**ConMed no. 03**: Daivobet® ointment is a combination product of calcipotriol and betamethasone used to treat psoriasis (= Medical history no. 2). The study participant applies 3 fingertip units of ointment per day on lesional skin. One “fingertip unit” is a strip of ointment, cream, gel, etc. from an individual’s distal interphalangeal joint of his or her index finger to the fingertip, corresponding to a length of approx. 2–3 cm. The route of administration is “12” (i.e., “Topical”) because the psoriatic skin is the target organ of Daivobet® ointment.

**ConMed no. 04**: The study participant applies a fentanyl TTS with a release rate of 25 µg fentanyl per hour on her skin for the treatment of chronic back pain (= Medical history no. 3). The patch is changed every 72 hours. In contrast to ConMed no. 03, the route of administration is not topical, but transdermal (i.e., “15”) because the skin itself is not the target organ, but serves for the absorption of fentanyl into the circulation.

**ConMed no. 05**: Triamcinolone acetonide was injected into the study participant’s left knee joint on 04-Mar-2019 due to activated gonarthrosis (= Medical history no. 4). Please note that the bold “03” in the start date has no particular meaning in terms of content, but was rather due to a technical issue of the biro used for documentation.

IU denotes international unit, ® registered trademark, TTS transdermal therapeutic system, x time, and XY dummy initials.


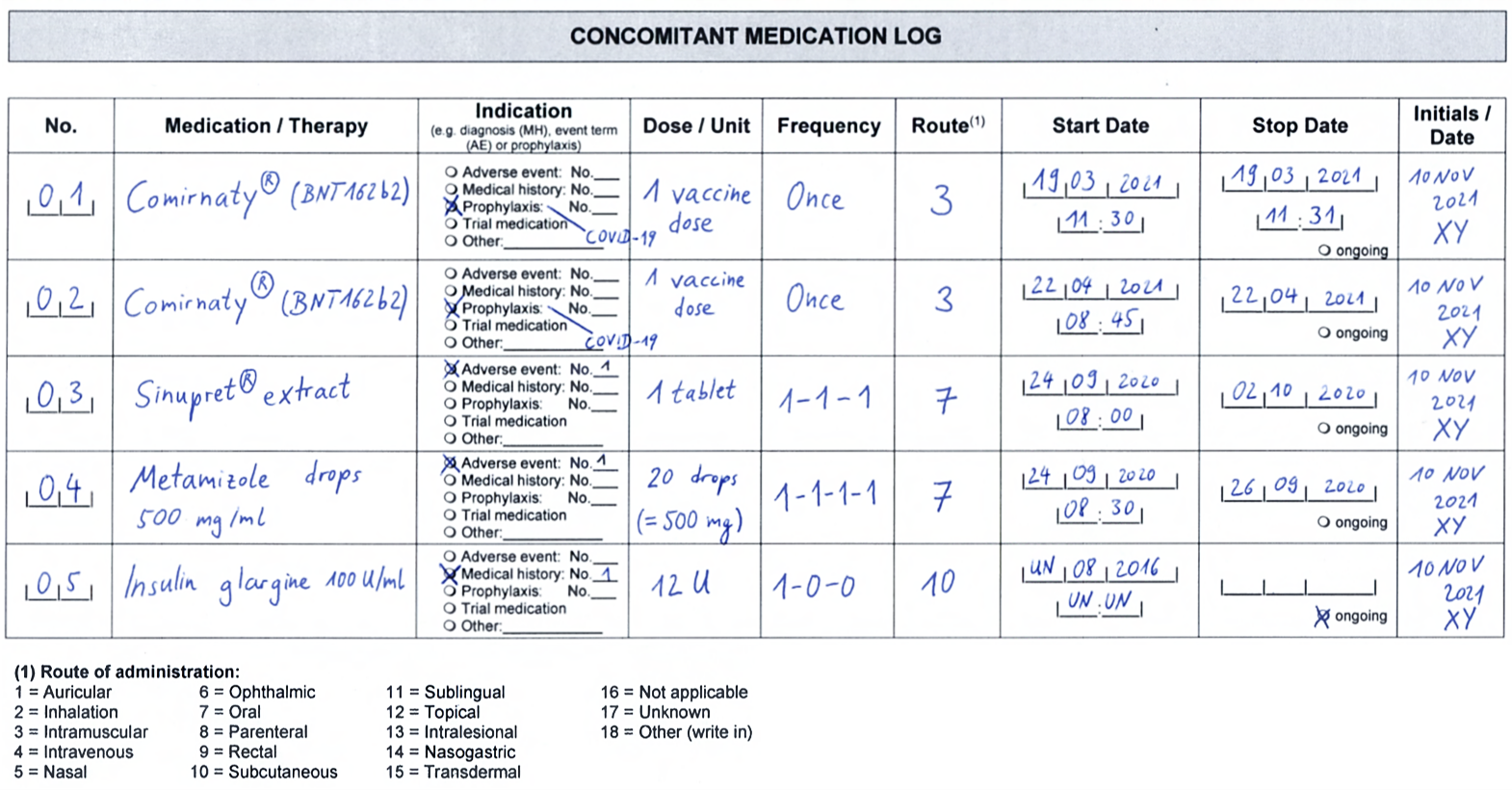


Fig. 6: Documentation of concomitant medication—third example

**ConMeds no. 01** **and no. 02**: Intramuscular administration of the COVID‑19 vaccine Comirnaty® (BNT162b2) on 19-Mar-2021 and on 22-Apr-2021. Please note that the occurrence of mild flu-like symptoms (e.g., headache, arthralgia, fever, etc.) is to be expected after these vaccinations. If such symptoms did not occur, a brief explicit note in the study participant’s file might be helpful for clarification.

**ConMed no. 03**: Sinupret® extract is a phytopharmaceutical containing five different herbal ingredients. Due to space limitations, it is preferred to only state the brand name instead of detailing all of the herbal ingredients of Sinupret® extract. Sinupret® extract was taken by the study participant against rhinosinusitis (= Adverse event no. 1).

**ConMed no. 04**: Twenty drops of metamizole (1 drop being equivalent to 25 mg) were taken four times a day by the study participant against headache as a symptom of rhinosinusitis (= Adverse event no. 1).

**ConMed no. 05**: Twelve units of insulin glargine are injected subcutaneously by the study participant every morning due to type‑2 diabetes mellitus (= Medical history no. 1).

COVID‑19 denotes coronavirus disease 2019, ® registered trademark, U unit, and XY dummy initials.

# Medical history

The medical history (MH) of a study participant comprises his or her diseases (both previous and ongoing), surgeries/interventions, and allergies. While allergies and ongoing diseases should be listed as completely as possible, only the clinically most relevant previous diseases and surgeries/interventions should be included into MH. The relevant timeframe for MH documentation is usually precisely defined in the clinical trial protocol. Previous diseases/surgeries/interventions that should be tabulated into the MH log include, but are not limited to:

- Diseases that required hospitalization
- Diseases that caused sequelae
- Sequelae of other diseases
- Major surgeries/interventions:
  - Surgical removal of organs: cholecystectomy, splenectomy, thyroidectomy, etc.
  - Organ transplantation: renal transplantation, liver transplantation, hematopoietic stem cell transplantation, etc.
  - Implantation of artificial joints (e.g., implantation of a total endoprosthesis of the hip joint)
  - Percutaneous coronary intervention (with or without stent implantation)
  - Implantation of a cardiac pacemaker/an ICD (implantable cardioverter defibrillator)

As a matter of fact, conditions that had existed before a study participant’s entry into a clinical trial, but that had been forgotten to be documented at enrollment, can still be added later to the MH log during the course of the trial (and in this case do not constitute AEs).

Furthermore, clinically suspicious findings detected during screening which have not previously been known to the study participant (e.g., cardiac murmurs detected for the first time during the screening examination at the study center) should be meticulously documented in the study participant’s MH log. If the finding is considered as potentially clinically relevant with regard to eligibility and the further course of the clinical trial, it appears prudent to initiate further examinations prior to enrollment. In the described scenario (i.e., cardiac murmurs auscultated for the first time during the screening examination), the investigator could, for example, initiate a transthoracic echocardiogram (TTE) and only proceed with enrollment if the TTE does not reveal any clinically relevant pathology.

## Documentation of medical history

An example of a complex medical history is discussed in **Fig. 7**.


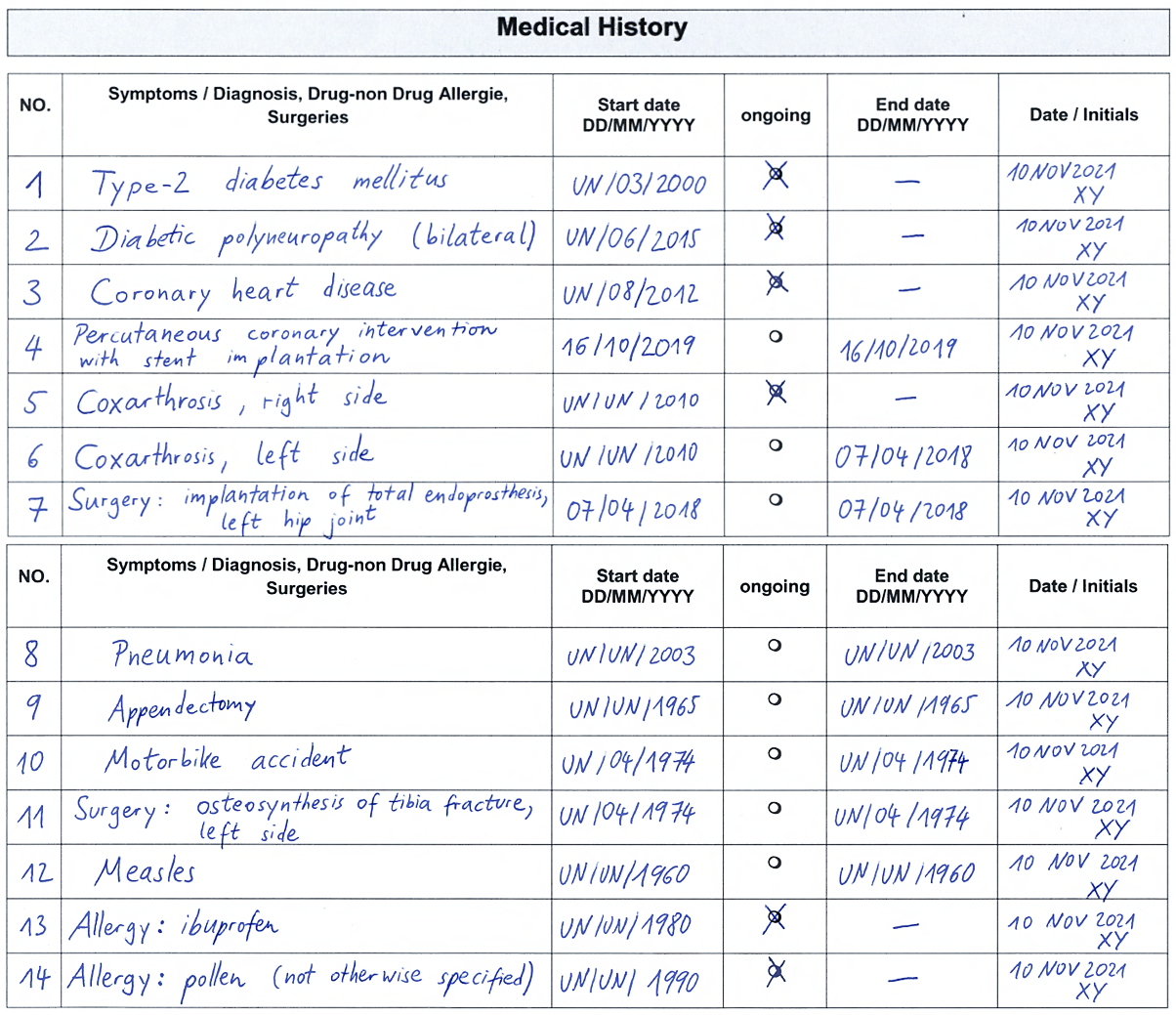


Fig. 7: Example of a complex medical history

In this example, the study participant’s MH comprises four chronic (and therefore ongoing) diseases, i.e.,

- type‑2 diabetes mellitus (= MH no. 1),
- diabetic polyneuropathy (= MH no. 2),
- coronary heart disease (= MH no. 3), and
- coxarthrosis of the right leg (= MH no. 5);

three previous (terminated) diseases, i.e.,

- coxarthrosis of the left leg (= MH no. 6),
- pneumonia (= MH no. 8), and
- measles (= MH no. 12);

four interventions/surgeries, i.e.,

- percutaneous coronary intervention (PCI) with stent implantation (= MH no. 4),
- implantation of a total endoprosthesis replacing the left hip joint (= MH no. 7),
- appendectomy (= MH no. 9), and
- osteosynthesis of a tibia fracture (= MH no. 11);

as well as two allergies, namely

- ibuprofen allergy (= MH no. 13) and
- pollen allergy (= MH no. 14).

Of note, the motorbike accident (= MH no. 10), which led to the tibia fracture in 1974, was documented separately. In contrast to the coxarthrosis of the right leg, which is still ongoing, the coxarthrosis of the left leg was *de facto* terminated by the surgical implantation of a total endoprosthesis. The date of the implantation of the total endoprosthesis hence represents the end date of the coxarthrosis of the left leg. Coronary heart disease, on the other hand, was only symptomatically treated with a PCI with stent implantation; the underlying atherosclerotic disease process, however, could not be terminated by this intervention. Therefore, coronary heart disease is still ongoing after 16-Oct-2019, the date of the PCI.

Type‑2 diabetes mellitus (= MH no. 1) and diabetic polyneuropathy (= MH no. 2) are patho­physiologically related, the latter being a sequela and indicator of the severity of the former. Therefore, diabetic polyneuropathy was documented separately in the study participant’s MH log.

If a study participant is unable to recollect certain medical details, such as the identity of the “pollen allergy” in this example, this can be documented as “not otherwise specified” (= MH no. 14).

# Quality control

## Quality control in clinical trials

Quality control is of paramount importance to ensure the adherence of clinical trials to GCP. It is the primary task of the trial monitor to safeguard the quality and reliability of the data generated within a clinical trial (chapter 8.1.1). Furthermore, different internal (chapter 8.1.2) and external (chapter 8.1.3) quality control mechanisms and checkpoints exist.

### Quality control via monitors

Quality control starts with diligently elaborated and consistently updated clinical trial protocol versions. For any amendment or major change to the trial protocol the study site and clinical team will be requested to verify protocol reading and training via signature and occasionally also via examinations.

The monitor, who acts on behalf of the sponsor of the clinical trial, regularly updates the study site about new versions of the trial protocol, the IB, etc., and checks the fulfillment of corresponding training measures, all of which will be documented and saved in the source data of the clinical trial.

If necessary, further electronic trainings will be requested via study-specific accounts to update the study sites on relevant new information or new functions of the eCRF. Such training measures often conclude with an examination. Passed examinations are confirmed with a certificate, which is saved in the source data and in the study site’s information technology (IT) system.

Monitors regularly visit the study site to check the source data as well as other aspects related to the clinical trial (e.g., SOPs, equipment, GCP certificates, and so forth).

### Internal quality control

Internal quality control takes place throughout the entire duration of the clinical trial. Several quality measures and checkpoints ensure the GCP-conform conduct of the clinical trial at the study site.

#### Worksheets

During data acquisition, the study staff works with worksheets specifically adapted to a specific study. This guarantees that all required and relevant information will be recorded during the study visits. Study visits are always conducted via the “four-eyes principle”, meaning that both a Study Nurse and an investigator are present, and that both of them review the data.

#### Cross-checking

Any data field on worksheets must be signed or initialed at least once (by the Study Nurse) or twice (by the Study Nurse and the investigator). We strongly recommend that source data are checked by a so-called “check-up twin” before datalock. The “check-up twin” usually is another Study Nurse who is only marginally involved with the clinical trial in question. This method ensures that the trial data are proofread by an unbiased person, which facilitates the detection of errors.

#### Quality management

Every study site has to provide a quality management (QM) system that implements the regular review and updating of SOPs. Standard operating procedures are created and reviewed by a multiprofessional team (including QM staff, Study Nurses, (sub)investigators, and PIs). The study personnel is required to confirm reading of updated or newly established SOPs with their signature and date of training. All SOP training logs are kept in an SOP binder which is stored at a central and easily accessible location at the study site. The SOP binder and the corresponding training logs are regularly checked by QM, monitors, and auditors.

### External quality control

External quality control is provided by means of queries (chapters 8.1.3.1 and 8.1.3.2), supervision by a medical monitor (MM) (chapter 8.1.3.3), a data and safety monitoring board (DSMB) (chapter 8.1.3.4), as well as via audits (chapter 8.1.3.6) and inspections (chapter 8.1.3.7).

#### Automated data queries

In addition to storage at the study site (via CRFs), data in clinical trials are uploaded on a trial-specific server (via eCRF), leading to “double back-up”. Information technology engineers program the eCRF so that it will automatically issue queries if the entered data is erroneous or logically inconsistent. The query will ask the user to rectify the incorrect entry. Unless the error is corrected, it is not possible to save the data or to move on to the next item.

#### Data management

Data that is entered into the eCRF is immediately reviewed and checked for inconsistencies by data management (DM) staff. In case of imprecise or inaccurate data, DM will issue a query which must be answered by a Study Nurse or an investigator.

#### Medical monitor

The MM (see also chapter 3) supervises the trial with regard to medical issues. Serious adverse events and AESIs have to be discussed with the MM, who can give medical advice or present the sponsor’s recommendations on specific issues. This ensures the highest possible safety and quality of medical treatment for the study participants and provides reassurance of the study staff with respect to possible protocol deviations.

#### Data and safety monitoring board

Many trials have external medical experts that form a multiprofessional and sometimes also multinational expert team, the DSMB (sometimes also referred to as data monitoring committee (DMC)). The DSMB meets at regular intervals and reviews and supervises SAEs and their handling. If the DSMB reaches the conclusion that there is imminent danger to an individual study participant, to other subjects in the trial, or to subjects in related trials (e.g., trials that investigate the same IMP), the DSMB can decide a hold of the trial and demand the implementation of further safety measures.

#### Update on suspected unexpected serious adverse reactions

The sponsor has to update the study sites regularly about the occurrence of SUSARs (chapter 5.7). The PIs and (sub)investigators have to read and sign the SUSAR reports provided by the sponsor to ensure the study participants’ continuous safety and well-being throughout the trial.

#### Audits

Audits take place on a regular basis at study sites to ensure that all study-related actions are adherent to GCP. This includes the review of SOPs, QM, the study personnel’s qualifications (including laboratory and pharmaceutical expertise), the equipment (i.e., devices, facilities, etc.) of the study site, and so forth. Audits are conducted by an auditor (chapter 3) on behalf of the sponsor of the clinical trial and are *“independent of and separate from routine monitoring”* [3].

#### Inspections

In contrast to audits (which are carried out by the sponsor or as internal QM measures), inspections are conducted by regulatory authorities. An inspection is an *“official review of documents, facilities, records, and any other resources that are deemed by the authority(ies) to be related to the clinical trial and that may be located at the site of the trial, at the sponsor’s and/or contract research organization’s (CRO’s) facilities, or at other establishments deemed appropriate by the regulatory authority(ies)”* [3]. In Germany, inspections can be carried out by the Federal Institute for Drugs and Medical Devices (*Bundesinstitut für Arzneimittel und Medizinprodukte* (BfArM)), by the Federal Institute for Vaccines and Biomedicines (*Paul-Ehrlich-Institut* (PEI)) as well as by the 16 different state authorities [20]. In the Netherlands, inspections can be carried out by the Healthcare and Youth Care Inspectorate (*Inspectie Gezondheidszorg en Jeugd* (IGJ)) of the Ministry of Health, Welfare and Sport (*Ministerie van Volksgezondheid, Welzijn en Sport* (VWS)). In addition, inspections can be conducted by supranational organizations (e.g., European Medicines Agency (EMA)) or even by agencies outside of the EU (e.g., United States Food and Drug Administration (FDA)).

## Quality control of the Students’ Guide

Important quality control aspects of the Students’ Guide to Documentation in Clinical Trials are version control (chapter 8.2.1), effective date (chapter 8.2.2), document history (chapter 8.2.3), page numbering (chapter 8.2.4), interdisciplinarity (chapter 8.2.5), multiprofessionality (chapter 8.2.6), internationality (chapter 8.2.7), digitalization (chapter 8.2.8), and accompanying educational activities (chapter 8.2.9), all of which are described in detail in the following chapters.

### Version control

As a rule, documents in clinical trials—such as the trial protocol, the IB, worksheets, but also the Students’ Guide to Documentation in Clinical Trials—are subject to version control. Version numbers x.**0** denote **final** versions whereas version numbers x.**1**, x.**2**, x.**3**, and so forth denote **draft** versions of a document. The very first draft of a document is usually referred to as version 0.1. The first final version of a document is commonly assigned the version number 1.0. Whenever a document needs to be updated, the next draft version is version 1.1. Once the updating process is completed, the next final version is version 2.0, and so on.

### Effective date

The effective date is an important information for the reader about the recency of a document. The effective date of this document, for example, is 21 June 2022; it can be found in the document history (chapter 1) as well as in the footer of every page. Date formatting aspects are discussed in detail in chapter 4.2.

### Document history

The document history keeps track of the changes that are implemented between different versions of a document. Moreover, the reason(s) for revision are specified in the document history, and the date when a new version of a document became effective (or becomes effective if the date lies in the future) is indicated. The document history is usually located at the beginning of a document; in the Students’ Guide it is displayed in chapter 1.

### Page numbering

In general, all documents in clinical trials contain page numbering. In contrast to the page numbering format used in the Students’ Guide, the preferred pagination style for documents in clinical trials is “Page x of n”, where x and n denote the current page and the overall number of pages, respectively.

### Interdisciplinarity

Interdisciplinarity is becoming increasingly important in human medicine as the (sub)specialization of medical disciplines constantly progresses. In clinical trials, it is indispensable that colleagues from different disciplines share their different expertise in order to achieve a common goal, that is, the successful conduct of a clinical study.

The Students’ Guide to Documentation in Clinical Trials is an interdisciplinary project in which specialists from clinical pharmacology, internal medicine, anesthesiology, neurology, and medical education cooperate.

### Multiprofessionality

The authors of the Students’ Guide to Documentation in Clinical Trials belong to different professions (physician, Study Nurse, medical student, voluntary scientific year) and different levels of medical hierarchy (Resident, Senior Physician, Head/Director). The joint first authors of the guide, Johannes Heck and Ann-Kathrin Rath, are Residents in clinical pharmacology and internal medicine, respectively. Katrin Wons is a Study Nurse at the Center for Clinical Trials of Hannover Medical School (MHH). Carsten Schumacher and Anna Kutschenko (who is Head of Clinical Operations at the Center for Clinical Trials of MHH) are Senior Physicians and specialists in anesthesiology and neurology, respectively. Nina Noltemeyer and Sarana Ulaganathan are medical students, while Luca J. Voßiek is conducting a voluntary scientific year (*Freiwilliges Wissenschaftliches Jahr*, FWJ) at MHH. Hemme Hijma is Senior Project Leader at the Centre for Human Drug Research (CHDR), Leiden. Jeroen van Smeden is Director Education at CHDR. Christoph Schröder is a specialist in internal medicine, Senior Physician, and Head of Pharmacovigilance in Clinical Trials at the Institute for Clinical Pharmacology, MHH. Dirk O. Stichtenoth is Director of the Institute for Clinical Pharmacology and Drug Commissioner of MHH. Heiner Wedemeyer is Director of the Department for Gastroenterology, Hepatology and Endocrinology of MHH. Christoph Schindler is Managing Head and Chief Medical Officer of the Center for Clinical Trials, MHH. Jacobus J. Bosch is Research Director Oncology at CHDR.

This demonstrates the broad spectrum of expertise and professional experience which significantly contributes to and ensures the quality of the Students’ Guide to Documentation in Clinical Trials.

### Internationality

The Students’ Guide to Documentation in Clinical Trials was developed at two sites in two countries: Hannover Medical School (MHH), Hannover, Germany and Centre for Human Drug Research (CHDR), Leiden, The Netherlands. This approach was chosen to enhance international collaboration in clinical trials. The synthesis of different national perspectives and particularities in a shared document improves the external validity, that is, the generalizability of the Students’ Guide to Documentation in Clinical Trials. Most notably, the medical dictionary (chapter 9) is provided in German, Dutch, and English. English was deliberately chosen as the primary language of the Students’ Guide to facilitate a more widespread use at other institutions and in other countries.

### Digitalization

A digital copy of the Students’ Guide to Documentation in Clinical Trials is accessible via the participating institutions’ IT infrastructures. Moreover, open-access publication of the guide in the *European Journal of Clinical Pharmacology* guarantees students, physicians, and other interested healthcare professionals from around the globe free access to this source of information.

### Educational activities

The Students’ Guide to Documentation in Clinical Trials forms an essential part of the training program of (medical) students working in clinical trials at MHH and CHDR. The guide serves both as a textbook and as a (quick-)reference work. It must be stressed that the Students’ Guide is not intended to replace practical training courses for students. On the contrary, the Students’ Guide has specifically been designed to complement practical educational activities.

# Medical dictionary German–English–Dutch

| **German**  (Medical term; colloquial term) | **English**  (Medical term; colloquial term) | **Dutch**  (Medical term; colloquial term) |
| --- | --- | --- |
| **Skin and skin appendages, including local reactions to injections/vaccinations** | | |
| Quaddel | Wheal | Kwaddel |
| Urtikariell; quaddelartig | Urticarial | Urticarieel; voorbijgaande roodheid |
| Erythem; Rötung | Erythema; redness | Erytheem; roodheid |
| Cicatrix; Narbe | Cicatrix; scar | Cicatrix; litteken |
| Narbenbildung | Cicatrization; scar formation | Littekenvorming |
| Hautausschlag | (Skin) rash (unspecific term; use only if a more precise term cannot be determined) | Huiduitslag |
| Exanthem (= plötzlich aufgetretener, großflächiger Hautausschlag) | Exanthema; skin eruption | Exantheem; rode huiduitslag (= plotselinge, uitgebreide huiduitslag) |
| Hautblase | Blister | Bulla; (huid)blaar |
| Bullös; blasenartig | Bullous | Bulleus; blaarvormig |
| Ekzem/Dermatitis; Hautentzündung | Eczema, dermatitis | Eczeem, dermatitis |
| Eiter | Pus | Pus |
| Pustel; (Eiter-)Pickel | Pustule | Pustel; puist |
| Abszess | Abscess | Abces; (nieuwgevormde) holte met pus |
| Ödem; Schwellung | Edema; swelling | Oedeem; (vocht)zwelling |
| **Otorhinolaryngology** | | |
| Verstopfte Nase | Nasal congestion | Verstopte neus |
| Rhinorrhoe/Rhinorrhö; laufende Nase | Rhinorrhea; runny nose | Rhinorrhoea/rinorroe; loopneus |
| Schnupfen | Rhinitis | Rhinitis; onsteking van neusslijmvlies |
| Fließschnupfen | Coryza | Nasofaryngitis/rinofaryngitis/coryza |
| Erkältung | Common cold | Verkoudheid |
| Epistaxis; Nasenbluten | Epistaxis | Epistaxis; neusbloeding/bloedneus |
| Pharyngitis; Halsentzündung, „roter Rachen“ | Pharyngitis; sore throat | Faryngitis; keelontsteking/keelpijn |
| Tonsillitis; Mandelentzündung | Tonsillitis | Tonsilitis; keel/neusamandelontsteking |
| Tonsillektomie; Mandelentfernung | Tonsillectomy | Tonsillectomie; keel/neusamandeloperatie |
| **Gastroenterology** | | |
| Reflux; Sodbrennen | Reflux/pyrosis; heartburn | Reflux; brandend maagzuur (een enkele keer) |
| Gastroösophageale Refluxerkrankung | Gastroesophageal reflux disease (GERD) | Gastro-oesofageale refluxziekte; brandend maagzuur (in meer chronische vorm) |
| Bauchschmerzen | Abdominal pain | Buikpijn |
| Diarrhoe/Diarrhö; Durchfall | Diarrhea | Diarree |
| Obstipation; Verstopfung | Constipation | Constipatie; verstopping |
| Flatulenzen; Blähungen | Flatulence | Flatulentie; winderigheid |
| Singultus; Schluckauf | Singultus; hiccup(s) | Singultus; de hik |
| Divertikulose | Diverticulosis | Diverticulosis; uitstulpingen in de dikke darm |
| Divertikulitis | Diverticulitis | Diverticulitis; onsteking van de uitstulpingen in de dikke darm |
| Appendizitis; Blinddarmentzündung^#^ | Appendicitis | Appendicitis; Blindedarmontsteking^#^ |
| Appendektomie; Blinddarmentfernung^#^ | Appendectomy | Appendectomie; Blindedarmoperatie^#^ |
| Gallenstein | Gallstone | Galsteen |
| Cholezystolithiasis; Gallensteinleiden | Cholecystolithiasis | Cholecystolithiasis; galsteenlijden |
| Cholezystitis; Gallenblasenentzündung | Cholecystitis | Cholecystitis; galblaasonsteking |
| Cholezystektomie; Gallenblasenentfernung | Cholecystectomy | Cholecystectomie; operatie om galblaas te verwijderen |
| Leistenhernie; Leistenbruch | Inguinal hernia | Hernia inguinalis; liesbreuk |
| **Neurology** | | |
| Kopfschmerzen | Headache | Hoofdpijn |
| Migräne | Migraine | Migraine |
| Migräneattacke | Migraine attack | Migraine aanval |
| Vertigo; „echter“ Schwindel | Vertigo | Vertigo; draaiduizeligheid (illusie van beweging) |
| Schwindel im Sinne von Benommenheit | Dizziness | Duizeligheid (in algemene zin) |
| Epilepsie | Epilepsy | Epilepsie; ‘vallende ziekte‘ |
| (Epileptischer) Anfall | (Epileptic) seizure | Epileptisch insult; epileptische aanval |
| Parästhesie/Sensibilitäts­störung; Missempfindung, „Ameisenlaufen“, „Kribbelgefühl“ | Paresthesia | Paresthesie; stoornis in gevoelsensatie |
| Schlaganfall | Stroke | Cerebro Vasculair Accident; beroerte |
| **Ophthalmology** | | |
| Verschwommenes Sehen | Blurred vision | Wazige visus; wazig zien |
| Reduzierte Sehschärfe | Reduced visual acuity | Verminderd scherpzien |
| Diplopie; Doppeltsehen, Doppelbilder | Diplopia | Diplopie; dubbelzien |
| Konjunktivale Hyperämie; Bindehautrötung | Conjunctival hyperemia, conjunctival reddening | Conjunctivale hyperemie/roodheid; rode ogen |
| Bindehautentzündung | Conjunctivitis | Conjunctivitis; bindvliesontsteking van het oog |
| Epiphora; Tränenträufeln | Epiphora | Epiphora; tranende ogen |
| Katarakt; Grauer Star | Cataract | Cataract, staar |
| Glaukom; Grüner Star | Glaucoma | Glaucoom; hoge oogdruk |
| Skotom; Gesichtsfeldeinschränkung, Gesichtsfeldverlust | Scotoma | Scotoom; blinde/zwarte plekken in het gezichtsveld |
| **Cardiovascular system** | | |
| Vorhofflimmern | Atrial fibrillation | Atriumfibrilleren; boezemfibrilleren |
| Vorhofflattern | Atrial fluttering | Atriumflutter; boezemflutter |
| Tachykardie; Herzrasen | Tachycardia | Tachycardie; te hoge hartslag |
| Herzinfarkt | Myocardial infarction | Myocardinfarct, hartinfarct |
| Koronare Herzkrankheit (KHK) | Coronary artery disease (CAD) | Coronair vaatlijden; kransslagaderaandoening |
| Periphere arterielle Verschlusskrankheit (pAVK); „Schaufensterkrankheit“ | Peripheral arterial occlusive disease (PAOD) | Perifeer arterieel vaatlijden |
| Karotisstenose | Carotid artery stenosis | Carotisstenose; vernauwde halsslagader |
| Ischämie; Minderdurchblutung | Ischemia | Ischemie; beperkte bloeddoevoer naar organen of weefsel |
| **Airways and lungs** | | |
| Dyspnoe/Atemnot; Kurzatmigkeit | Dyspnea; shortness of breath | Dyspneu; kortademigheid |
| Husten | Cough(ing) | Aspecifieke hoestklachten; hoesten |
| Reizhusten | Dry cough | Droge hoest |
| Bronchitis | Bronchitis | Bronchitis; ontsteking van de luchtpijptak |
| **Systemic complaints** | | |
| Fieber | Fever; pyrexia | Hyperthermie; koorts |
| Schüttelfrost | Chills | Koude rilling |
| Müdigkeit | Tiredness | Vermoeidheid |
| Unwohlsein | Malaise | Malaise; algehele staat van ongemak |
| Abgeschlagenheit | Fatigue | Verminderde energie |
| Kraftlosigkeit | Asthenia | Krachteloos |
| Schwäche | Adynamia | Zwakte |
| Hyperhidrosis; vermehrtes/übermäßiges Schwitzen | Hyperhidrosis | Hyperhidrosis; overmatig zweten |
| Nachtschweiß | Night sweats | Nachtzweten |
| Muskelschmerzen | Myalgia | Myalgie; spierpijn |
| Muskuloskelettale Schmerzen, Schmerzen des Bewegungsapparats | Musculoskeletal pain | Musculoskeletale pijn; spier- en beenderpijnen |
| Nackenschmerzen | Neck pain | Nekpijn |
| Rückenschmerzen | Back pain | Rugpijn |
| Gelenkschmerzen | Arthralgia | Arthralgie, gewrichtspijn |
| Gliederschmerzen | Limb pain | Pijn in ledematen |
| Gewichtsverlust | Weight loss | Gewichtsverlies |
| Gewichtszunahme | Weight gain | Gewichttoename |
| **Drug formulations** | | |
| Tablette | Tablet | Tablet |
| Kapsel | Capsule | Capsule |
| Lutschtablette | Lozenge | Pastille |
| Kautablette | Chewable tablet | Kauwtablet |
| Brausetablette | Effervescent tablet | Bruistablet |
| Creme | Cream | Creme |
| Salbe | Ointment | Zalf |
| Ampulle | Ampoule | Ampul |
| Durchstechflasche | Vial | Flacon |
| Hub (Inhaler) | Puff | Pufje (inhalator) |
| Nasenspray | Nasal spray | Neusspray |
| Sprühstoß (Nasenspray) | Application/spray/puff (nasal spray) | Pufje (neusspray) |
| Spritze | Syringe | Spuit |
| Kanüle | Cannula | Canule |
| **Good Clinical Practice** | | |
| Gute Klinische Praxis | Good Clinical Practice | Good Clinical Practice |
| Unerwünschtes Ereignis (UE) | Adverse event (AE) | Adverse event (AE) |
| Schwerwiegendes unerwünschtes Ereignis (SUE) | Serious adverse event (SAE) | Serious adverse event (SAE) |
| Schwerwiegendheit; schwerwiegend | Seriousness; serious | Ernst; ernstig |
| Schwere; schwer | Severity; severe | Hevigheid; heftig |
| Erwartbarkeit | Expectedness | Verwachting |
| Erwartet/unerwartet | Expected/unexpected | Onverwacht/verwacht |
| Verdachtsfall einer schwerwiegenden unerwarteten Nebenwirkung | Suspected unexpected serious adverse reaction (SUSAR) | Een vermoeden van een onverwachte ernstige bijwerking |
| Informierte Einwilligung | Informed consent | Informed consent; geïnformeerde toestemming |
| Probandeneinwilligung (Dokument) | Informed consent form (ICF) | Toestemmingsverklaring |
| Studienformular | Case report form (CRF) | Case report form (CRF) |
| Elektronisches Studienformular | Electronic case report form (eCRF) | Electronic case report form (eCRF) |
| Prüferinformation/Prüfarzt­broschüre | Investigator’s brochure (IB) | Investigator’s brochure (IB) |
| Fachinformation | Summary of product characteristics (SmPC) | Samenvatting van de product kenmerken |

^#^This term is commonly used by laypeople, even though medically imprecise.

# References

1. European Medicines Agency. Clinical Trials Regulation. Available at: <https://www.ema.europa.eu/en/human-regulatory/research-development/clinical-trials/clinical-trials-regulation>. Accessed January 27, 2022

2. Official Journal of the European Union (2014) Regulation (EU) No 536/2014 of the European Parliament and of the Council of 16 April 2014 on clinical trials on medicinal products for human use, and repealing Directive 2001/20/EC. Available at: <https://eur-lex.europa.eu/legal-content/EN/TXT/PDF/?uri=CELEX:32014R0536&from=EN>. Accessed January 27, 2022

3. International Council for Harmonisation of Technical Requirements for Registration of Pharmaceuticals for Human Use (ICH) Guideline for good clinical practice E6(R2). Available at: <https://www.ema.europa.eu/en/ich-e6-r2-good-clinical-practice#current-version---revision-2-section>. Accessed November 18, 2021

4. European Medicines Agency. National competent authorities (human). Available at: <https://www.ema.europa.eu/en/partners-networks/eu-partners/eu-member-states/national-competent-authorities-human>. Accessed June 17, 2022

5. European Commission (2011) Detailed guidance on the collection, verification and presentation of adverse event/reaction reports arising from clinical trials on medicinal products for human use (CT-3). Available at: <https://eur-lex.europa.eu/LexUriServ/LexUriServ.do?uri=OJ:C:2011:172:0001:0013:EN:PDF>. Accessed June 17, 2022

6. United States Department of Health and Human Services (2017) Common Terminology Criteria for Adverse Events (CTCAE) Version 5.0. Available at: <https://ctep.cancer.gov/protocolDevelopment/electronic_applications/docs/CTCAE_v5_Quick_Reference_5x7.pdf>. Accessed January 03, 2022

7. Medical Dictionary for Regulatory Activities. Available at: <https://www.meddra.org/>. Accessed January 03, 2022

8. United States Department of Health and Human Services, Food and Drug Administration (FDA), Center for Drug Evaluation and Research (CDER), Center for Biologics Evaluation and Research (CBER) (2011) Guidance for Industry—E2F Development Safety Update Report. Available at: <https://www.fda.gov/media/71255/download>. Accessed January 26, 2022

9. Council for International Organizations of Medical Sciences (CIOMS) (2005) Management of Safety Information from Clinical Trials—Report of CIOMS Working Group VI. Available at: <https://cioms.ch/wp-content/uploads/2017/01/Mgment_Safety_Info.pdf>. Accessed January 26, 2022

10. Bonhoeffer J, Kohl K, Chen R, Duclos P, Heijbel H, Heininger U, Jefferson T, Loupi E (2002) The Brighton Collaboration: addressing the need for standardized case definitions of adverse events following immunization (AEFI). Vaccine 2002; 21(3–4): 298–302. doi: S0264410X02004498 [pii]

11. Petousis-Harris H (2020) Assessing the Safety of COVID-19 Vaccines: A Primer. Drug Saf 2020; 43(12): 1205–1210. doi: 10.1007/s40264-020-01002-6

12. Safety Platform for Emergency vACcines (SPEAC) (2021) SO2-D2.1.3 Priority List of COVID-19 Adverse events of special interest—Part 2. Update for COVID-19 complications other than Long COVID. Available at: <https://brightoncollaboration.us/wp-content/uploads/2021/11/SO2_D2.1.3_COVID-19_AESI-update_V1.0_Part-2_09Nov2021.pdf>. Accessed January 26, 2022

13. Safety Platform for Emergency vACcines (SPEAC) (2021) SO2-D2.1.2 Priority List of COVID-19 Adverse events of special interest: Quarterly update December 2020. Available at: <https://brightoncollaboration.us/wp-content/uploads/2021/01/SO2_D2.1.2_V1.2_COVID-19_AESI-update_V1.3.pdf>. Accessed January 26, 2022

14. Kaehler KC, Hassel JC, Heinzerling L, Loquai C, Thoms KM, Ugurel S, Zimmer L, Gutzmer R, committee on “Cutaneous Adverse Events” of the German Working Group for Dermatological Oncology (Arbeitsgemeinschaft Dermatologische Onkologie, ADO) (2020) Side effect management during immune checkpoint blockade using CTLA-4 and PD-1 antibodies for metastatic melanoma—an update. J Dtsch Dermatol Ges 2020; 18(6) :582–609. doi: 10.1111/ddg.14128

15. Heinzerling L, de Toni E, Schett G, Hundorfean G, Zimmer L (2019) Checkpoint Inhibitors. Dtsch Arztebl Int 2019; 116: 119–126. doi: 10.3238/arztebl.2019.0119

16. Mai K, Fassnacht M, Führer-Sakel D, Honegger JB, Weber MM, Kroiss M (2021) The Diagnosis and Management of Endocrine Side Effects of Immune Checkpoint Inhibitors. Dtsch Arztebl Int 2021; 118: 389–396. doi: 10.3238/arztebl.m2021.0143

17. Boos G (2019) Präklinische Dokumentation und Investigator’s Brochure. Available at: <https://www.bfarm.de/SharedDocs/Downloads/DE/Service/Termine-und-Veranstaltungen/ringvorlesungen/2019_Winter/Boos_191119.pdf?__blob=publicationFile&v=2>. Accessed January 18, 2022

18. Le J. Drug Administration. MSD Manual Consumer Version. Available at: <https://www.msdmanuals.com/home/drugs/administration-and-kinetics-of-drugs/drug-administration?query=Drug%20Absorption>. Accessed December 30, 2021

19. Lampert A, Haefeli WE, Seidling HM (2019) Information Gaps in Package Inserts Cause Insufficient Patient Information on Correct Handling of Transdermal Patches. Dtsch Med Wochenschr 2019; 144(5): e36–e41. doi: 10.1055/a-0722-7419

20. Witte B (2017) GCP Inspektionen klinischer Prüfungen. Available at: <https://www.bfarm.de/SharedDocs/Downloads/DE/Service/Termine-und-Veranstaltungen/ringvorlesungen/2016_Winter/GCP1701.pdf?__blob=publicationFile&v=4>. Accessed January 18, 2022
